# Single-cell transcriptomic comparison of tubular segment maturation in advanced human *in vitro* kidney models

**DOI:** 10.1016/j.isci.2026.116053

**Published:** 2026-05-22

**Authors:** Carla Pou Casellas, Fjodor A. Yousef Yengej, Carola M.E. Ammerlaan, Gisela G. Slaats, Maarten B. Rookmaaker, Marianne C. Verhaar, Hans Clevers

**Affiliations:** 1 Hubrecht Institute, Royal Dutch Academy of Science (KNAW) and University Medical Center Utrecht (UMCU), Utrecht, the Netherlands; 2 Department of Nephrology and Hypertension, University Medical Center Utrecht (UMCU), Utrecht, the Netherlands

**Keywords:** Computational bioinformatics, Nephrology, Stem cells research, Systems biology, Tissue engineering, Transcriptomics

## Abstract

*In vitro* kidney models play a crucial role in the study of renal development, (patho)physiology, drug development, and potential applications in bioartificial kidneys. Human *in vitro* kidney models have recently advanced significantly with the development of (induced) pluripotent stem cell ((i)PSC)-derived organoids, tissue/urine-derived tubuloids, and iPSC organoid-derived tubuloids (iPSCod tubuloids). However, challenges remain in understanding degree and diversity of differentiation and each model’s benefits. Here, we compared the transcriptomic profiles of kidney tubular epithelial cell types across several advanced three-dimensional (3D) models with physiological *in vivo* profiles (adult and fetal). Our selected models included differentiated kidney tissue-derived tubuloids, iPSCod tubuloids, and four iPSC-derived organoid models from different protocols. Our findings highlight distinct transcriptional profiles, the presence of diverse adaptive cell states, and differences in maturation marker expression among models. This study offers insights into the characteristics of state-of-the-art kidney models and guidance for researchers choosing suitable models for their investigations.

## Highlights

• Integration of scRNA-seq datasets enables benchmarking of advanced kidney models
• First scRNA-seq characterization of iPSC organoid-derived tubuloids (iPSCod tubuloids)
• Identification of model-specific cell identities, adaptive states, and maturation levels
• Distal tubule, connecting tubule, and intercalated cells are scarce in all included models

## Graphical abstract

**Figure:**
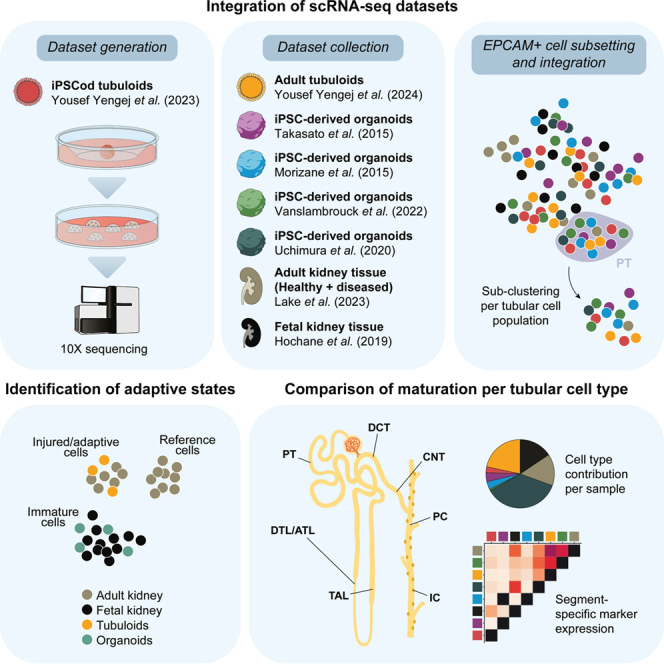

## Introduction

Organoids are multicellular 3D structures that can be generated from different starting cell populations, including embryonic stem cells, induced pluripotent stem cells (iPSCs), and adult stem/progenitor cells. PSC-derived kidney organoids were established in the past decade, with foundational protocols that are still widely used nowadays.^1^^,^^2^^,^^3^^,^^4^ This system represents an intricate miniature model of the nephron, including an organized tubular epithelium, mesenchyme, and endothelium. However, cellular maturity in PSC-derived organoids is mostly representative of late fetal kidney stages.^5^^,^^6^ To enhance maturation and reduce the presence of off-target populations, several optimized protocols have succeeded the originals.^7^^,^^8^^,^^9^^,^^10^^,^^11^^,^^12^^,^^13^

In 2019, we reported the generation of primary tissue- and urine-derived kidney tubuloids.^14^ Tubuloids are cystic structures comprised of multiple kidney epithelial cell populations. This reductionist system does not generate off-target cell populations and has advantages in terms of long-term expansion, genetic stability, and overall resemblance to physiological tissue expression. However, this model also has limitations including the lack of podocytes and non-epithelial cell types, and the absence of a nephron-like morphology as observed in PSC-derived organoids. Recently, we developed an improved differentiation protocol for tubuloids, which allows for expression of functional markers expressed in collecting duct (CD) and loop of Henle (LOH) cells.^15^

Bridging the culture of kidney organoids and tubuloids, we also showed the generation of so-called iPSC organoid-derived kidney tubuloids (iPSCod tubuloids). This hybrid approach is based on differentiated iPSC-derived kidney organoids (d7+18), which are dissociated, and subsequently cultured and differentiated following a tubuloid culture protocol. This allows for improved cell expansion and results in a reduction of immature and off-target cell populations.^16^

Given the diverse advanced kidney models and the ongoing optimization of their differentiation, it is becoming increasingly challenging for researchers to keep up with each model’s characteristics and marker expression profile. Therefore, we conducted a comprehensive comparison of single-cell transcriptomic profiles among a selection of advanced kidney models relative to the *in vivo* human nephron, both at an adult and fetal stage.^17^^,^^18^ Since tubuloids generally recapitulate an injured/regenerative *in vivo* state, we selected the adult kidney tissue dataset generated by Lake et al. (2023), which is comprised of both healthy/reference kidneys and diseased kidneys.^17^ As exemplary *in vitro* models, we chose kidney tissue-derived tubuloids differentiated using Yousef Yengej’s protocol,^14^^,^^15^ iPSCod tubuloids^16^—which we have sequenced here for the first time—and four iPSC-derived kidney organoid models obtained through different protocols: two pioneer protocols developed by Takasato et al. (2015)^3^ and Morizane et al. (2015)^4^^,^^19^ and two more recent protocols by Vanslambrouck et al. (2022) and Uchimura et al. (2020), in which differentiation was directed toward proximal tubule (PT) and CD, respectively^7^^,^^10^ (Table 1).

**Table 1:** Datasets used in this study

Model	Protocol	N° of samples	Line	Ethical approval	Seq. method	GEO access. n°	Reference
Tubuloids	Yousef Yengej et al.^15^	1 sample of >100 tubuloids	#138H	yes	10× scRNA-seq	GEO: GSE206538	Yousef Yengej et al.^15^
iPSCod tubuloids	Yousef Yengej et al.^16^	1 sample of >100 tubuloids	iPSC15	yes	10× scRNA-seq	GEO: GSE303210	This work
iPSC-derived organoids–––	Takasato et al.^3^	1 pool of 65 organoids (2 reps)–	BJFF.6–	NR–	DropSeq scRNA-seq–	GEO: GSE118184–	Wu et al.^19^–
	Morizane et al.^4^						
	Vanslambrouck et al.^10^	4 pooled organoids (4 reps)	CRL1502.C32	cell line derived from WS1 CRL-1502^TM^ fibroblasts (ATCC)	10× scRNA-seq	GEO: GSE184928	Vanslambrouck et al.^10^
	Uchimura et al.^7^	NR	BJFF.6	NR	10× scRNA-seq	GEO: GSE131086	Uchimura et al.^7^
Adult kidney tissue	Lake et al.^17^	58 reference (35 donors) and 52 diseased tissues (36 patients)	N/A	yes	10× scRNA-seq	GEO: GSE183276	Lake et al.^17^
Fetal kidney tissue	Hochane et al.^18^	1 kidney (w16)	N/A	yes	10× scRNA-seq	GEO: GSE114530	Hochane et al.^18^

NR, not reported.

For a comprehensive transcriptomic comparison of the models, we evaluated each major renal segment individually. Given that neither tubuloids nor iPSCod tubuloids contain glomerular cells, we decided to focus exclusively on the tubular epithelial cell types (i.e., PT, descending [DTL] and ascending thin limb [ATL], thick ascending limb [TAL], distal convoluted tubule [DCT], connecting tubule [CNT], principal cells [PCs], and intercalated cells [ICs]). For each major cell type, we first explored cell heterogeneity by identifying subpopulations. Using the original adult kidney tissue annotations, we could discern where injured (i.e., both (mal)adaptive/regenerative and degenerative) cells clustered and subsequently assign states to cells in the models. The level of maturation in each model was thereafter evaluated by exploring transcriptional congruence with adult kidney tissue.

This single-cell atlas of advanced *in vitro* kidney models and *in vivo* tissue has enabled the identification of both *in vivo*-resembling and non-physiological cell types, differences in adaptive states, and an in-depth scrutiny of gene expression landscapes across the studied models. This study provides a resource for researchers seeking guidance in selecting the most appropriate model for their specific objectives, whether focused on developmental processes, disease modeling, drug testing studies, or the development of bioartificial kidneys. We also provide a web-based explorer for free exploration of the tubular epithelial comparison data, accessible through https://kidneymodelcomparison.shinyapps.io/shinyapp/.

## Results

### Integration of transcriptomic profiles of advanced kidney models with human adult and fetal primary tissue

**Figure 1:**
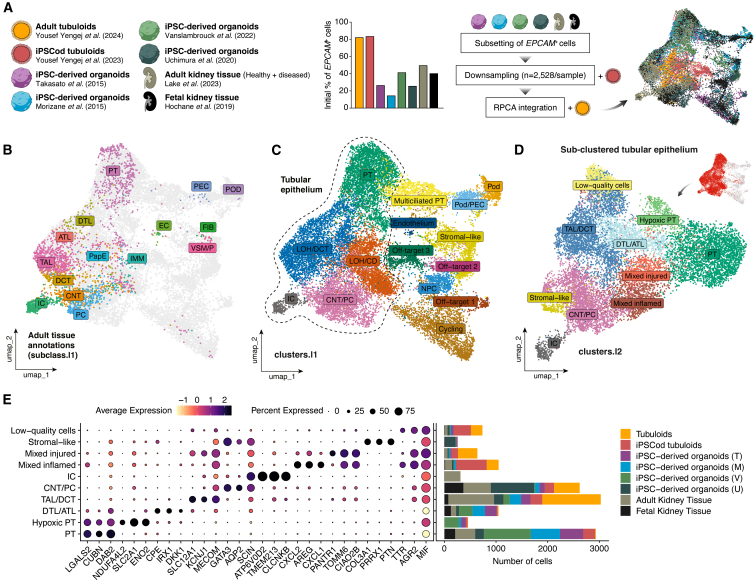
Single-cell atlas of state-of-the-art *in vitro* human kidney models integrated with adult and fetal kidney tissue datasets (A) Diagram illustrating the samples included in this study, the percentage of *EPCAM*+ cells in each sample, and the processing strategy for integration. (B) UMAP plot of the integrated atlas depicting the original annotations of adult kidney tissue-derived cells [subclass.l1 from Lake et al. (2023)^17^]. (C) UMAP plot of the integrated atlas showing identified clusters (clusters.l1). (D) UMAP plot of the sub-clustered tubular epithelium populations with deeper clustering annotations (clusters.l2). (E) Dot plot depicting top three DEGs per cluster, including the cell contribution of each sample to the individual clusters. See also Figure S1 and S2.

To compare cell type abundance and transcriptome of adult tissue-derived kidney tubuloids, iPSCod tubuloids, iPSC-derived kidney organoids, and adult and fetal human kidney tissue, we integrated all datasets into a single atlas. To circumvent batch effects, we integrated the same number of cells per sample. Since the tubuloid dataset contained much fewer cells than the rest, we first carried out enrichment of epithelial cells in iPSC-derived organoid and tissue datasets by subsetting *EPCAM*+ cells (which only represented up to 50% of cells). Subsequently, all datasets were downsampled to the number of cells sequenced in tubuloids (2,528 cells) and integrated using reciprocal principal-component analysis (RPCA). This yielded an atlas of 20,224 cells (Figure 1A). All cell number metrics upon subsetting and downsampling can be found in Table S1.

Unbiased clustering led to the identification of 15 clusters. Using the available metadata from the adult kidney tissue dataset (Figure 1B), we could easily discern broad kidney epithelial cell populations, cycling cells, and some endothelial and stromal contamination (Figure 1C). In addition, we identified nephron progenitor cells (NPCs) (*TMEM100*+, *CITED1*+),^20^^,^^21^ three off-target populations, and a PT subpopulation that we referred to as “multiciliated PT.” The top differentially expressed genes (DEGs) in each cluster (level 1) can be found in Figure S1A and Data File 1. To determine whether the different samples/batches were properly mixed upon integration, we computed NEST-Scores. The high (∼0) scores in most clusters reflected good sample mixing. Lower scores were mostly observed in protocol-specific cell types, including ICs and off-target 3 cells (Figure S1B).

Off-target 1 (*MSX1*+, *RSPO3*+) and 2 (*RTN1*+, *STMN4*+) cells were mostly derived from iPSC-derived organoid protocols, while the off-target 3 cluster (*RFX6*+, *GATA4*+) was primarily derived from iPSCod tubuloids (Figures S1A and S1C).

Multiciliated PT cells were predominantly observed in iPSC-derived organoid samples and fetal kidney tissue (Figure S1D). These cells were characterized by high expression levels of multiciliated cell markers (e.g., *CFAP126*, *ARMC3*, *CAPSL*, and *FOXJ1*^22^^,^^23^^,^^24^) and showed co-expression with bona fide PT markers such as cubilin (*CUBN*)^25^ (Figures S1E and S1F). Gene Ontology (GO) term enrichment analysis confirmed that pathways involving cilium movement and organization were predominant in these cells (Figure S1G). The presence of multiciliated cells in kidney PT *in vivo* has been confirmed in human fetal tissue and injured adult tissue.^26^^,^^27^

Given the high prevalence of non-renal or immature epithelial cell types, we sub-clustered the kidney tubular epithelium compartment to increase cluster resolution (Figure 1D). All samples showed broad cell distribution over the clusters (Figure S2A), and both NEST-Score and kBET indicated adequate sample mixing. Once again, lower scores were however observed for protocol/sample-specific cell types (e.g., ICs and hypoxic PT) (Figures S2B and S2C).

Using the original adult kidney tissue annotations (Figure S2D) and exploring DEGs per cluster (Figure 1E; Data S2), we identified seven populations: PT (*LGALS2*+, *CUBN*+), DTL/ATL (*CPE*+, *IRX1*+), TAL/DCT (*SLC12A1*+, *MECOM*+), CNT/PC (*GATA3*+, *AQP2*+), IC (*ATP6V0D2*+, *CLCNKB*+), and mixes of inflamed (*CXCL2*+, *AREG*+) and injured (*PANTR1*+, *TOMM6*+)^28^^,^^29^ cells. Besides these, one cluster showed scarce expression of relevant cell type-specific markers (Figure 1E), along with lower feature counts/cell (Figure S2E), hence why it was annotated as “low-quality cells.” We also observed two clusters containing minimal cells derived from adult kidney tissue, namely stromal-like cells (*GATA3*+ *PTN*+), mostly arising from Uchimura’s organoids, and what we annotated as “hypoxic PT” cells (Figures 1D and 1E). Around 60% of cells in the hypoxic PT cluster came from organoids derived using the Vanslambrouck protocol (Figure S2F). Hypoxic PT cells were characterized by high expression of markers such as *NDUFA4L2*, *SLC2A1*, and *ENO2*,^30^^,^^31^^,^^32^ which were partially co-expressed with the mature PT marker *CUBN* (Figures S2G and S2H). GO term enrichment analysis validated the activation of hypoxia-related pathways in this cluster (Figure S2I). All top DEGs in the hypoxic PT cluster (e.g., *SLC2A1*, *ENO2*, and *LGI4*) have been previously observed in cultured iPSCod tubuloids in response to hypoxia.^33^

### Transcriptomic comparison of PT cells in advanced kidney models

**Figure 2:**
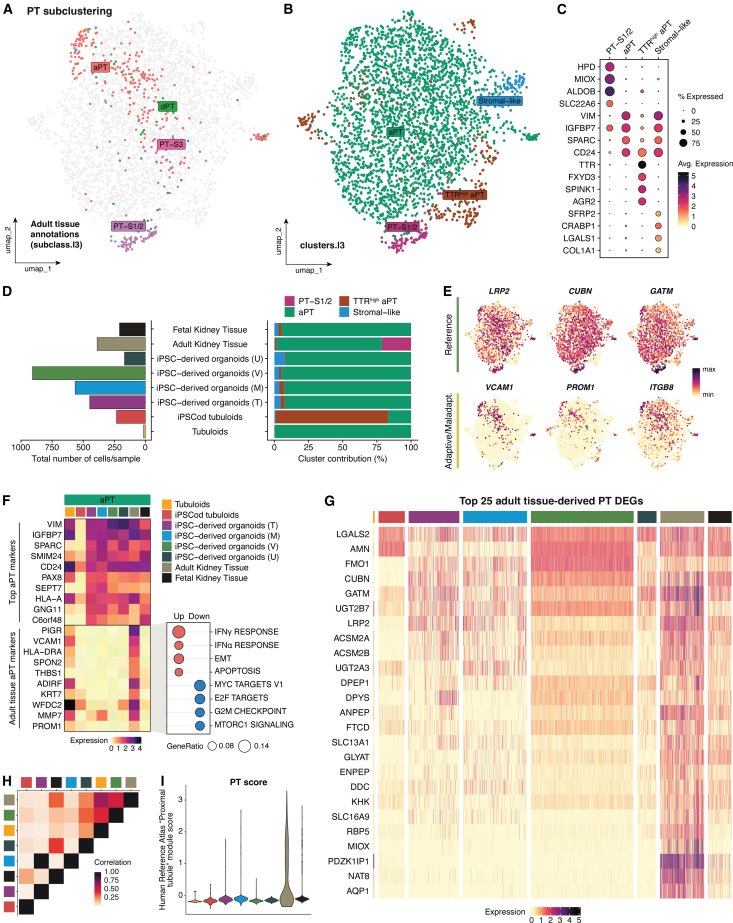
Transcriptomic profile comparison of proximal tubule (PT) cells across *in vitro* kidney models and primary human tissue (A) UMAP plot of the sub-clustered PT population highlighting the original annotations of adult kidney tissue-derived cells [subclass.l3 from Lake et al. (2023)^17^]. (B) UMAP plot of the sub-clustered PT population showing identified clusters (clusters.l3). (C) Dot plot depicting top four DEGs per cluster. (D) Total PT cell numbers and cluster contribution percentage in each sample. (E) Feature plots of reference and adaptive/maladaptive PT markers. (F) Heatmap showing average expression levels of general aPT markers and adult tissue aPT markers across samples, along with gene set enrichment analysis in adult tissue-derived aPT cells. (G) Heatmap of the top 25 DEGs in adult tissue-derived PT cells plotted across samples. (H) Heatmap illustrating correlation in average expression across samples in PT cells. (I) PT score per cell based on validated markers from the Human Reference Atlas’ Anatomical Structures, Cell Types, and Biomarkers (ASCT+B) tables. All heatmaps show normalized expression values, not row- or column-scaled. See also Figure S3.

Upon subsetting of the PT cluster from the tubular epithelial atlas, we could identify several PT subpopulations. Using the annotations of the kidney tissue dataset, we located a distinct PT-S1/2 cluster (*ALDOB*+, *SLC22A6*+) and adaptive PT (aPT) cells (Figures 2A and 2B). Notably, native cells belonging to the last segment (PT-S3) were present in minimal numbers and could therefore not be properly subclustered. We also identified a *TTR*^high^ aPT population (*FXYD3*+, *AGR2*+) and an aPT cluster expressing stromal markers (e.g., *LGALS1*, *COL1A1*), which was annotated as “stromal-like” (Figures 2B and 2C; Data S3).

In agreement with their aim toward a PT-enhanced organoid model, the highest numbers of (a)PT cells came from organoids generated using Vanslambrouck’s protocol. The lowest PT cell numbers (<20) came from tubuloids, which was expected given that Yousef Yengej’s differentiation protocol aimed at maturation of distal tubular segments^15^ (Figure 2D).

When assessing cluster abundance per sample, we observed very similar patterns between fetal kidney tissue and all four iPSC-derived kidney organoid models. The majority of cells in these samples (>75%) were considered aPT but also contained some stromal-like cells. The adult kidney tissue sample primarily contained aPT cells, but around 20% of cells were PT-S1/2. Finally, tubuloids and iPSCod tubuloids had some aPT cells, but iPSCod tubuloids mostly contributed to *TTR*^high^ aPT cells (Figure 2D). Notably, aPT DEGs were related to increased epithelial-to-mesenchymal transition (EMT) and Wnt signaling, which are commonly attributed to kidney injury.^34^ In contrast, *TTR*^high^ aPT DEGs showed enrichment in cholesterol homeostasis and estrogen response (Figure S3A).

In the original publication of the adult kidney tissue dataset, Lake et al. presented short lists of genes reflecting a reference (healthy) or (mal)adaptive phenotype.^17^ By plotting those genes across our (a)PT clusters, we observed broad expression of reference PT genes *LRP2*, *CUBN*, and *GATM* (Figure 2E). Expression of (mal)adaptive markers *VCAM1*, *PROM1*, and *ITGB8*, in contrast, was mostly limited to a subset of cells in the aPT cluster belonging to the adult kidney tissue sample. To investigate the difference between adult tissue- and non-tissue-derived aPT cells, we first looked at the expression of the top aPT cluster DEGs across samples. All samples, except for iPSCod tubuloids, showed high expression of adaptive markers such as *VIM*, *CD24*, and *PAX8*. When exploring the top DEGs in adult tissue-derived aPT among all aPT cells, we found that most cells additionally expressed reported maladaptive/failed-repair PT markers (e.g., *PIGR*, *VCAM1*, and *MMP7*),^35^^,^^36^^,^^37^^,^^38^^,^^39^^,^^40^ and these were only additionally observed in tubuloid-derived cells (Figures 2F and S3B). MSigDb enrichment analysis indicated an increase in inflammatory response and apoptosis in adult-tissue-derived aPT cells, while the remaining aPT cells showed activation of MYC and MTORC1 signaling pathways (Figure 2F).

Other canonical PT injury markers, such as *HAVCR1* (KIM-1), *SOX9*, and *PAX2*,^38^^,^^41^^,^^42^ were found expressed in all aPT clusters and were especially abundant in tubuloids (Figure S3C).

After characterizing PT subpopulations, we went on to investigate expression levels of maturation/functional markers across samples. As a general indication of maturation, we first evaluated expression of the top 25 DEGs found in adult-tissue-derived PT cells across all samples (independent of clustering). Cells from iPSC-derived kidney organoids cultured using Vanslambrouck’s protocol showed the most generalized expression of several of these PT-specific markers (e.g., *CUBN*, *ACSM2A*, and *ANPEP*), followed by the other three organoid models and iPSCod tubuloids (Figure 2G). To provide a more accessible list of adult PT DEGs, Figures S3D–S3F depict expression of top transporters, enzymes, and other genes in all samples. Importantly, the expression of relevant solute carriers such as *SLC6A19* (B(0)AT1), *SLC22A6* (OAT1), and *SLC5A2* (SGLT2) was scarce in all *in vitro* models (Figure S3D). We also assessed expression of the top transcriptional regulators found in adult PT cells (*MAF*, *HNF4A*, *TFEC*, and *HNF4G*) and found highest generalized expression in Vanslambrouck’s organoid-derived PT cells. iPSCod tubuloid PT cells also showed high, abundant expression of the majority of these genes (Figure S3G), which has also been demonstrated using immunofluorescence.^16^ Notably, however, these factors in iPSCod tubuloids did not induce expression of many PT-specific genes but rather more general target genes (e.g., *HNF4A-ALB* and *MAF-ANPEP*) that are also associated with metabolic processes in other organs such as the liver and intestine.^43^^,^^44^

The few tubuloid aPT cells present barely expressed any mature PT marker. However, since they shared an adaptive signature with adult kidney tissue-derived aPT cells, their transcriptome was found to be highly correlated with that of adult tissue. After tubuloids, PT cells from Vanslambrouck’s organoids, fetal kidney tissue, and Uchimura’s organoids had the most similar average expression levels to adult kidney tissue (Figure 2H). Despite the low correlation in average expression compared to adult tissue, using a set of PT-specific validated markers from the Human Reference Atlas (HRA),^45^ we observed that Takasato’s and Morizane’s organoids had the highest PT scores (Figure 2I).

### Transcriptomic comparison of TAL/DCT cells in advanced kidney models

**Figure 3:**
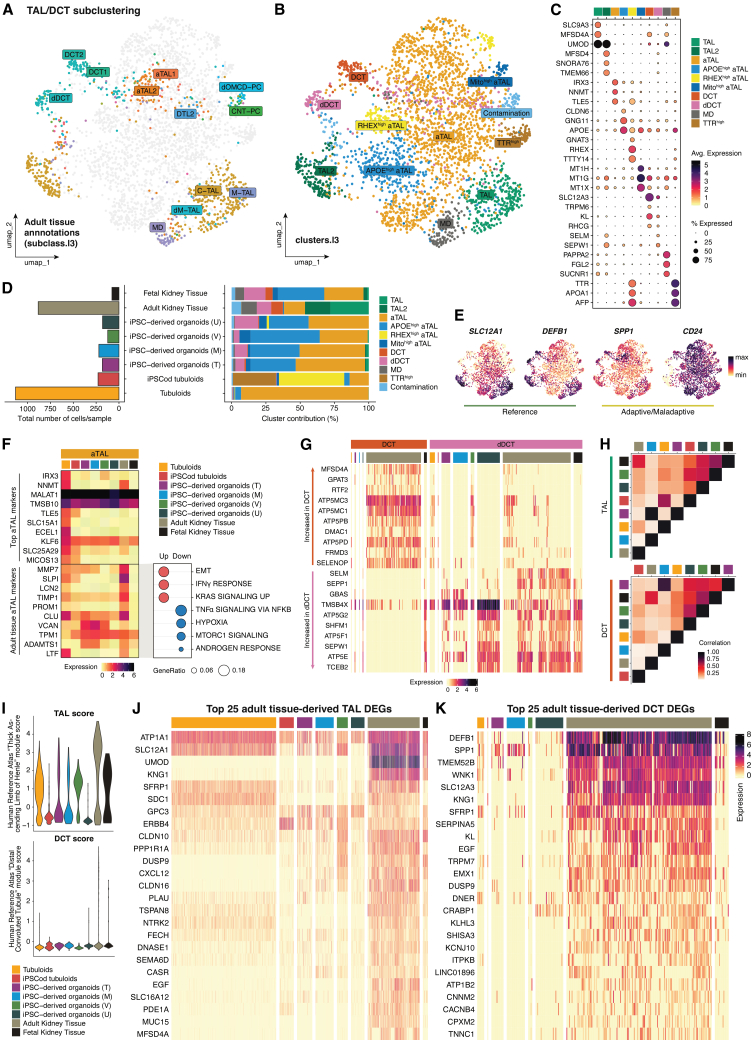
Transcriptomic profile comparison of thick ascending limb (TAL) and distal convoluted tubule (DCT) cells across *in vitro* kidney models and primary human tissue (A) UMAP plot of the sub-clustered TAL/DCT population highlighting the original annotations of adult kidney tissue-derived cells [subclass.l3 from Lake et al. (2023)^17^]. (B) UMAP plot of the sub-clustered TAL/DCT population showing identified clusters (clusters.l3). (C) Dot plot depicting top three DEGs per cluster. (D) Total TAL/DCT cell numbers and cluster contribution percentage in each sample. (E) Feature plots of reference and adaptive/maladaptive TAL markers. (F) Heatmap showing average expression levels of general aTAL markers and adult tissue aTAL markers across samples, along with gene set enrichment analysis in adult tissue-derived aTAL cells. (G) Heatmap of markers increased in DCT versus dDCT plotted in all samples. (H) Heatmap illustrating correlation in average expression across samples in both TAL and DCT cells. (I) TAL and DCT scores per cell based on validated markers from the Human Reference Atlas’ Anatomical Structures, Cell Types, and Biomarkers (ASCT+B) tables. (J and K) Heatmaps of the top 25 DEGs in adult tissue-derived TAL and DCT cells plotted across samples. All heatmaps show normalized expression values, not row- or column-scaled. See also Figure S4.

In the subsetted TAL/DCT cluster, the differently annotated adult tissue-derived cells orderly clustered together (Figure 3A). Based on that distribution, we could identify several sub-populations: TAL (predominantly cortical TAL [C-TAL]), which was divided into TAL (*SLC9A3*+, *UMOD*+) and TAL2 (*UMOD*+, *TMEM66*+); adaptive TAL (aTAL) (*IRX3*+, *NNMT*+); DCT (*SLC12A3*+, *TRPM6*+); damaged DCT (dDCT); and macula densa (MD) (*PAPPA2*+, *FGL2*+). Moreover, we noted the presence of three aTAL clusters that were annotated as *APOE*^high^ aTAL, *RHEX*^high^ aTAL, and mito^high^ aTAL; a *TTR*^high^ population and some PC-derived contamination (Figures 3B and 3C; Data S4).

The major part of TAL/DCT cells came from tubuloids and adult kidney tissue. The adult kidney tissue dataset was primarily comprised of TAL, TAL2, aTAL, DCT, dDCT, and MD. Once again, a similar cell composition was observed between fetal kidney tissue and iPSC-derived organoids. These samples contained comparable numbers of aTAL and *APOE*^high^ aTAL cells and some dDCT cells. Tubuloids mostly contributed to aTAL cells, and while iPSCod tubuloids also had some aTAL cells, the majority of cells were *RHEX*^high^ aTAL, and *TTR*^*h*igh^ (Figure 3D). Genes identified by Lake et al. (2023) as reference (*SLC12A1* and *DEFB1*) and (mal)adaptive TAL (*SPP1* and *CD24*)^17^ were broadly expressed in all (a)TAL clusters (Figure 3E).

Since aTAL was the only altered cluster containing adult tissue-derived cells, we next explored whether *in vitro* model-derived cells in that cluster could accurately recapitulate the *in vivo* adaptive state. Top aTAL DEGs were mostly dictated by tubuloid-derived aTAL cells, but some were shared across samples (e.g., *MALAT1*, *TMSB10*, and *KLF6*). Adult tissue-derived aTAL cells expressed high levels of adaptive markers such as *MMP7*, *TIMP1*, and *CLU*, which were also expressed in the other samples. Distinctively, certain tissue aTAL markers including *LCN2* (NGAL), *PROM1* (CD133), and *LTF*^46^^,^^47^ were additionally only found in tubuloid-derived cells. Adult tissue aTAL DEGs indicated an increase in EMT and interferon gamma (IFN-γ) response, while the rest of aTAL cells showed enriched hypoxia and MTORC1 signaling (Figure 3F).

Despite the fact that organoid-derived cells clustered together with *in vivo* MD cells, top MD markers *PAPPA2*, *FGL2*, *SUCNR1*, and *CA4* were only relevantly expressed in adult and fetal kidney tissue-derived cells (Figure S4A). This suggests that (re-)differentiation of MD cells *in vitro* is currently lacking.

Besides MD, both tubuloids and organoids contained dDCT cells. To understand the differences between dDCT and DCT cells, we plotted some of the top markers in each population across samples. We noticed that iPSC-derived organoids obtained using either the Takasato, Morizane, or Uchimura protocols lacked expression of healthy DCT markers in both DCT and dDCT and had tissue-like expression of *in vivo* dDCT markers (e.g., *ATP5G2* and *ATP5E*). Tubuloids and Vanslambrouck’s organoids contained less dDCT cells, but these cells actually expressed markers enriched in the DCT cluster, such as *ATP5MC3* and *ATP5PB*, and lacked dDCT marker expression (Figure 3G). To further confirm that these ATPases were differentially expressed in DCT and dDCT cells, we assessed co-expression of the DCT marker *SLC12A3* (NCC) with either *ATP5MC3* or *ATP5L*. The majority of DCT cells co-expressed *SLC12A3* and *ATP5MC3*, whereas co-expression of *SLC12A3* with *ATP5L* was limited to dDCT cells. Moreover, when assessing the expression of DCT (*ATP5MC3*, *ATP5MC1*, and *ATP5PD*) and dDCT markers (*ATP5G2*, *ATP5E*, and *SEPP1*) across physiological cell states, DCT markers were expressed in all cell states (adaptive, degenerative, and reference), while dDCT markers were mostly enriched in degenerative cells (Figures S4B and S4C).

To survey the extent of TAL and DCT maturation in the models, we first separated all TAL/aTAL from DCT/dDCT clusters to explore correlation in average expression. In TAL clusters, adult kidney tissue-derived cells shared a more similar transcriptome to fetal tissue cells, followed by lower correlations with tubuloids and both Vanslambrouck’s and Uchimura’s organoids. As for DCT cells, fetal tissue, followed by tubuloids, showed the highest correlation to the adult tissue transcriptome (Figure 3H). TAL identity scores based on HRA markers appeared highest in tubuloids and Vanslambrouck’s organoids but were generally high in all models. Contrarily, DCT scores were overall very low, with only some cells in tubuloids and Uchimura’s organoids showing higher score (Figure 3I).

Next, we evaluated expression of the top 25 *in vivo* TAL and DCT markers across samples (Figures 3I and 3J). As seen in Figure 3I, some genes involved in electrolyte regulation were abundantly expressed in most models, including *ATP1A1* (Na^+^K^+^ATPase), *SLC12A1* (NKCC2), and *CLDN10*. Presence of Na^+^K^+^ATPase and NKCC2 has also been confirmed at the protein level in some of the models.^7^^,^^10^^,^^15^^,^^16^ Numerous TAL-specific transporters and enzymes were present in *in vitro* models (Figures S4D and S4E). However, other relevant TAL markers, such as the immune-related *UMOD* and *GP2*^48^ were virtually absent in both tubuloids and organoids (Figure S4F).

DCT cells in the models studied weakly expressed top tissue-derived DCT markers, especially transporters and channels (Figures 3J and S4G–S4I). Tubuloids and Uchimura’s iPSC-derived organoids were the only samples containing a few *SLC12A3*+ and *TRPM7*+ cells. NCC staining has been confirmed in FAF-differentiated tubuloids.^15^ Other DCT-specific genes involved in the regulation of electrolyte homeostasis, such as *KLHL3* and *KCNJ10*,^49^^,^^50^ were absent in all *in vitro* models and fetal kidney tissue (Figure 3J). The top transcription factors (TFs) found in TAL and/or DCT cells were variably expressed across models. Overall, the highest and most abundant expression of several factors (e.g., *IRX2*, *TFAP2B*, *ESRRG*, and *LHX1*^51^) was observed in Vanslambrouck’s iPSC-derived organoids (Figure S4J).

### Transcriptomic comparison of CNT/PCs in advanced kidney models

**Figure 4:**
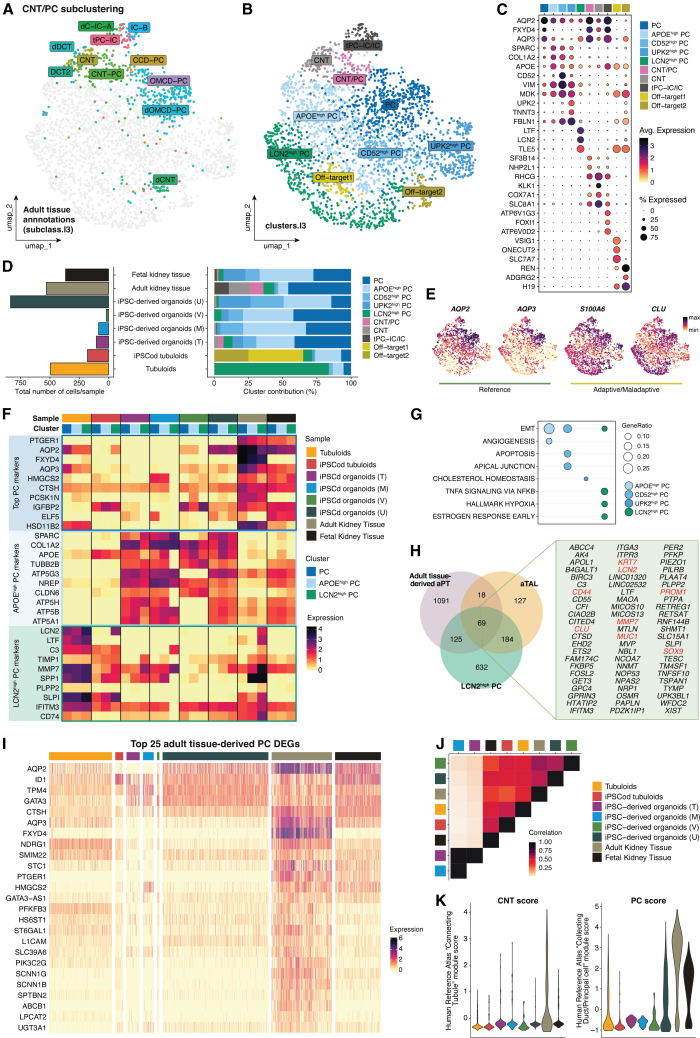
Transcriptomic profile comparison of connecting tubule (CNT) and principal cells (PC) across *in vitro* kidney models and primary human tissue (A) UMAP plot of the sub-clustered CNT/PC population highlighting the original annotations of adult kidney tissue-derived cells (subclass.l3 from Lake et al. (2023)^17^). (B) UMAP plot of the sub-clustered CNT/PC population showing identified clusters (clusters.l3). (C) Dot plot depicting top three DEGs per cluster. (D) Total CNT/PC cell numbers and cluster contribution percentage in each sample. (E) Feature plots of reference and adaptive/maladaptive PC markers. (F) Heatmap showing average expression levels of top PC, *APOE*^high^ PC, and *LCN2*^high^ PC markers across samples per cluster. (G) Comparison of gene set enrichment analysis between adaptive PC populations. (H) Venn diagram depicting the overlapping DEGs in mature adaptive populations across the nephron. In red are highlighted common adaptive genes upregulated upon kidney injury. (I) Heatmap of the top 25 DEGs in adult tissue-derived PC plotted across samples. (J) Heatmap illustrating correlation in average expression across samples in both CNT and PC. (K) CNT and PC scores per cell based on validated markers from the Human Reference Atlas’ Anatomical Structures, Cell Types, and Biomarkers (ASCT+B) tables. All heatmaps show normalized expression values, not row- or column-scaled. See also Figure S5.

The last cluster subsetted from Figure 1D for extensive characterization was the CNT/PC cluster. The increased resolution enabled the identification of distinct populations using adult kidney tissue annotations as reference (Figure 4A): PC (mostly medullary) (*AQP2*+, *AQP3*+), CNT/PC (*AQP2*+, *RHCG*+), CNT (*KLK1*+, *SLC8A1*+), and transitioning PC-IC (tPC-IC)/IC (*AQP2*+, *FOXI1*+). Additionally, we identified several adaptive PC populations (*APOE*^high^ PC, *CD52*^high^ PC, *UPK2*^high^ PC, and *LCN2*^high^ PC) and off-target cells (*VSIG*1+, *H19*+) (Figures 4B and 4C; Data S5). In line with the intended differentiation in the models studied, tubuloids and iPSC-derived organoids obtained using the Uchimura protocol contributed the most CNT/PCs, besides adult and fetal kidney tissue. The lowest number of cells came from Vanslambrouck’s organoids (Figure 4D).

Cluster composition within iPSC-derived organoids and fetal kidney tissue was once again very comparable. These samples primarily contained PC, *APOE*^high^ PC, *CD52*^high^ PC, and *UPK2*^high^ PC. Adult kidney tissue contributed to PC, CNT/PC, CNT, and tPC-IC/IC and to a lesser extent, *APOE*^high^ PC and *LCN2*^high^ PC. Lastly, tubuloids chiefly contained *LCN2*^high^ PC, whereas iPSCod tubuloids mostly had *APOE*^high^ PC and off-target cells (Figure 4D). Of note, off-target 2 cells expressed both *AQP3* and *REN*. Even though no tissue-derived cells were found in this cluster, we cannot exclude the possibility of them representing (pro)renin-producing CD cells.^52^ Adult tissue reference (*AQP2* and *AQP3*) and (mal)adaptive (*S100A6* and *CLU*) markers^17^ were broadly expressed across all clusters (Figure 4E).

Since the main PC populations found *in vivo* appeared to be PC, *APOE*^high^ PC, and *LCN2*^high^ PC, we went on to investigate how these clusters differ from each other both in primary tissue and *in vitro* models. By plotting the top DEGs per cluster across samples, we noticed that all samples generally maintained a shared signature across clusters. Tubuloids and iPSCod tubuloids expressed *LCN2*^high^ PC markers (e.g., *C3* and *SLPI*), while iPSC-derived organoids and fetal kidney tissue expressed markers of *APOE*^high^ PC (e.g., *NREP* and *CLDN6*), independent of clustering. Adult kidney tissue, in contrast, did show enrichment of *LCN2*^high^ PC markers, including *LTF* and *MMP7* in the *LCN2*^high^ PC cluster, indicating that this is the *bona fide* adaptive PC population in adult tissue (Figure 4F).

*APOE*^high^ and *CD52*^high^ PC cells showed activation of EMT and apoptotic processes, respectively. Conversely, *LCN2*^high^ PC cells had increased tumor necrosis factor alpha (TNF-α) signaling and hypoxia (Figure 4G). Since we observed similar signatures and enriched processes between “mature” adaptive cells across nephron cell types, we investigated the genes co-expressed in adult tissue-derived aPT (i.e., *VCAM1*^high^), aTAL, and *LCN2*^high^ PC. Sixty-nine genes were found to be expressed in the three populations, including *CD44*, *KRT7*, *LCN2*, *PROM1*, and *SOX9* (Figure 4H). This confirms that all major tubular kidney cells acquire a conserved set of adaptive genes during injury, which has also been reported in previous studies.^37^^,^^47^^,^^53^^,^^54^

To explore PC maturation in *in vitro* models compared to kidney tissue, we again computed the top DEGs in adult tissue-derived PCs and assessed expression in each sample. Only tubuloids and iPSC-derived organoids obtained using the Uchimura protocol showed abundant expression of the hormone-regulated *AQP2* and *AQP3* (Figure 4I), which has been confirmed at the protein level.^7^^,^^15^ Some PC markers were found in all models, including *SLC38A1* (SNAT1), *CTSH*, and *KRT19*. However, expression of transport regulators such as *FXYD4*, *PTGER1*, and *ABCB1* (P-gp) was minimal or absent in the models (Figures 4I and S5A–S5C). PC TFs (e.g., *GATA3*, *ID1*, and *HES1*) were present in most models (Figure S5D). Correlation of average expression confirmed that all models shared a similar PC transcriptomic pattern with that of adult kidney tissue, except for Takasato and Morizane’s organoids (Figure 4J).

CNT cells were barely found in *in vitro* models. Nonetheless, we looked at expression of top adult-tissue-derived CNT markers across all clusters in each sample. The CNT-specific gene *CALB1* (calbindin) was mostly found in Morizane and Uchimura’s iPSC-derived organoids, while *SLC8A1* (NCX1) was present but lowly expressed in all models. Other mature CNT markers such as *RHCG* and *KL* (klotho) were almost exclusively found in tissue (Figure S5E). When assessing both CNT and PC identity scores in CNT/PC cells of each sample, we observed similar (low) CNT scores, while PC scores were higher in tubuloids and Uchimura’s organoids (Figure 4K).

### Transcriptomic comparison of less abundant tubular cell populations in advanced kidney models

Lastly, we gave some attention to tubular cell clusters less abundant in our dataset: DTL/ATL, IC, and mixed inflamed and injured cells.

**Figure 5:**
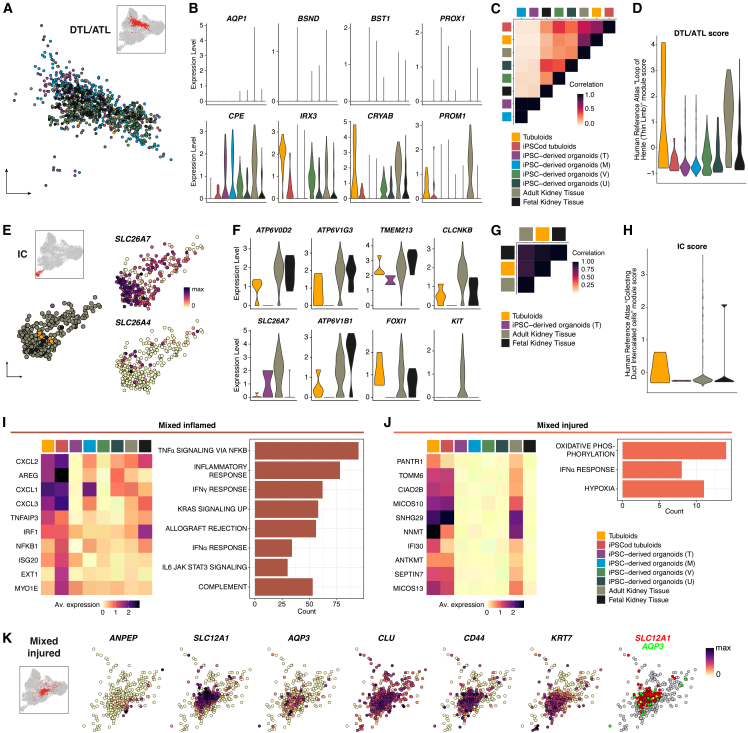
Transcriptomic profile comparison of descending/ascending thin limb (DTL/ATL), intercalated cells (IC), and mixed cell populations across *in vitro* kidney models and primary tissue (A) UMAP plot of the DTL/ATL cluster showing sample composition. (B) Violin plots of the expression of common and computed DTL/ATL DEGs in each sample. (C) Heatmap illustrating correlation in average expression across samples in the DTL/ATL cluster. (D) DTL/ATL score per cell based on validated markers from the Human Reference Atlas’ Anatomical Structures, Cell Types, and Biomarkers (ASCT+B) tables. (E) UMAP plot of the IC cluster showing sample composition along with relative expression of the IC-A marker *SLC26A7* and the IC-B marker *SLC26A4*. (F) Violin plots showing expression of top IC(-A) DEGs across samples. (G) Heatmap illustrating correlation in average expression across samples with >3 ICs. (H) IC score per cell based on validated markers from the Human Reference Atlas’ Anatomical Structures, Cell Types, and Biomarkers (ASCT+B) tables. (I and J) Heatmap depicting average expression levels of top mixed inflamed and mixed injured DEGs in each sample, together with hallmark pathways found to be activated in each cluster. (K) Feature plots showing relative expression of markers present in the mixed injured cluster. Heatmaps show normalized expression values, not row- or column-scaled.

The DTL/ATL cluster contained a homogeneous mix of all samples (Figure 5A). Previously reported DTL/ATL markers such as *AQP1*, *BSND*, *BST1*, and *PROX1*^55^^,^^56^^,^^57^ were virtually absent in all samples, including adult kidney tissue. Instead, most samples showed increased expression of other thin limb markers including *CPE*, *IRX3*, *CRYAB*, and *PROM1*,^58^^,^^59^^,^^60^ which might be indicative of an injured/adaptive phenotype (Figure 5B). Despite the low abundance of DTL/ATL cells in tubuloids, the transcriptome of these cells—followed by iPSCod tubuloids—most highly correlated with that of adult kidney tissue (Figure 5C). Moreover, tubuloid DTL/ATL cells showed the highest DTL/ATL score (Figure 5D).

Being a lowly abundant cell type, the IC cluster was represented by a few cells, the majority coming from adult kidney tissue. Moreover, most cells were found to be IC-A (*SLC26A7*+) rather than *SLC26A4*+ IC-B (Figure 5E). Similar to DTL/ATL cells, tubuloids had very few cells annotated as IC, but these cells expressed mature IC markers such as *ATP6V0D2*, *CLCNKB*, and *FOXI1*.^61^ Besides tubuloids, only Takasato’s organoids contained scarce ICs but lacked expression of several functional markers (Figure 5F). Correlation of average expression confirmed that tubuloid- and both fetal and adult tissue-derived ICs share a similar transcriptomic profile (Figure 5G). Of note, Takasato’s organoids could not be included in this correlation analysis given that they only had two ICs. HRA validated IC markers were also confirmed in the few tubuloid cells but overall lowly expressed (Figure 5H).

Finally, we found two populations with mixed identities that primarily contained cells coming from tubuloids and iPSCod tubuloids (Figure 1E). Mixed inflamed cells expressed high levels of chemokines (e.g., *CXCL1*, *CXCL2*, and *CXCL3*) across all samples except for Takasato and Vanslambrouck’s organoids. Other immune response regulators (e.g., *TNFAIP3*, *IRF1*, and *NFKB1*), however, were generally found in all samples. Gene set enrichment analysis corroborated the activation of inflammatory pathways via NFKB in these cells (Figure 5I). This inflammatory phenotype has also been recently reported in PT cells of patients with unilateral ureteric obstruction.^62^ In mixed injured cells, the top DEGs, which included markers of oxidative stress (*NNMT*),^63^ senescence (*SNHG29*),^64^ and apoptosis (*SEPTIN7*),^65^ were exclusively present in tubuloids, iPSCod tubuloids, and adult kidney tissue (Figure 5J). This mixed injured cluster contained cells expressing segment-specific genes (e.g., PT [*ANPEP*+], TAL [*SLC12A1*+], and PC [*AQP3*+]), as well as adaptive markers (e.g., *CLU*, *CD44*, and *KRT7*). Notably, some cells in this cluster co-expressed markers of different segments (e.g., *SLC12A1* and *AQP3*) (Figure 5K). Such mixed-identity phenotype has been previously observed both *in vivo* and *in vitro.*^15^^,^^66^

### Model identities and modeling capabilities remain consistent upon capped downsampling

Since we downsampled all datasets to 2,528 cells (being tubuloids the limiting factor), there was a risk of losing statistical power and diversity from larger datasets. To investigate this, we performed a sensitivity analysis using less stringent, capped downsampling and evaluated whether conclusions hold. All samples were downsampled to contain up to 5,000 cells, which allowed the inclusion of 100% *EPCAM*+ cells coming from tubuloid, iPSCod tubuloid, Morizane’s organoid, Uchimura’s organoid, and fetal kidney tissue datasets. The cell numbers for the other three samples (Takasato and Vanslambrouck’s organoids and adult kidney tissue) were significantly increased, providing better coverage (Figure S6A; Table S2). Aided by the adult kidney tissue dataset annotations and by exploring DEGs per cluster, we found back all clusters that had been previously identified in both epithelial and tubular epithelial atlases (Figures S6B and S6C). Batch effects were once again mainly observed in the IC and off-target cell clusters (Figure S6D). Distribution of samples across clusters remained similar, as did the top DEGs per cluster (Figure S6E).

As a simplified way to validate our conclusions on cell type modeling capabilities of the models, for each major segment we compared cell numbers per sample and a segment-specific identity score based on the top 25 DEGs in adult kidney tissue cells. Overall, the relative abundance of the samples in each cluster remained similar upon capped downsampling (Figures S6F–S6J). The only exception were tubuloids, which, upon addition of more cells from other datasets, had some cells re-classified from “TAL/DCT” and “CNT/PC” to “mixed injured/inflamed” cells (Figures S6H and S6I). The calculated identity scores per cell type also appeared comparable after capped downsampling (Figures S6F–S6J). Of note, all samples showed a lower DCT score upon capped downsampling because more TAL (and MD) cells were included in that joint TAL/DCT cluster, thus “diluting” DCT cells (Figure S6H). In conclusion, using a different dataset downsampling strategy did not affect the main conclusions of this study.

## Discussion

To utilize *in vitro* 3D kidney platforms for applications such as disease modeling, drug testing, and regenerative therapies, it is important to understand the unique benefits of each system and how these compare to primary kidney tissue. In this study, we integrated the transcriptome of state-of-the-art *in vitro* kidney models alongside adult and fetal primary kidney tissue to assess comparability. Integration of all datasets enabled us to study similarities and differences in transcriptomic profile of each major nephron epithelial cell type in the models versus both *in vivo* healthy and injured kidney cells as reference. While a recent study by Nunez-Nescolarde et al. (2025) compared human kidney model transcriptomes using bulk RNA sequencing,^33^ here we leverage a single-cell resolution to uncover cellular heterogeneity and proportions, identify rare cell types and non-physiological states, and ultimately assess model suitability per cell type. Other studies have previously compared iPSC protocols (with adult and fetal tissue) using scRNAseq^19^^,^^67^^,^^68^; the present work also includes tubuloids and iPSCod tubuloids.

Irrespective of the cell type, cells in a healthy/reference state were almost exclusively found in adult kidney tissue. Tubuloid-derived cells showed an adaptive state characteristic of injured mature kidney, and iPSC-derived kidney organoids replicated immature states (i.e., adaptive states that were also observed in fetal kidney tissue). This is in line with their nature in being bottom-up (iPSC-derived organoid) and top-down (tubuloid) *in vitro* culture strategies.

It is noteworthy that iPSCod tubuloids contained a lot of cells in altered epithelial states, which we considered off-targets. The majority of cells in iPSCod tubuloids expressed a set of markers including *TTR*, *APOA1*, *AGR2*, *FXYD3*, and *AFP*, which are more commonly observed in (immature) hepatocytes and intestinal cells.^69^^,^^70^^,^^71^^,^^72^ This highlights the importance of transcriptome profiling of heterogeneous cultures at a single-cell level. The nature of TTR^high^ cell clusters (e.g., TTR^high^ aPT) remains unclear. However, they may reflect a partially off-target cell population rather than a physiological adaptive state. Being derived from iPSC organoids, which contain fetal-like and also non-mesodermal cells, a three-dimensional (3D) culture high in Wnt and FGF might promote (*trans*-)differentiation into other epithelial lineages.

**Figure 6:**
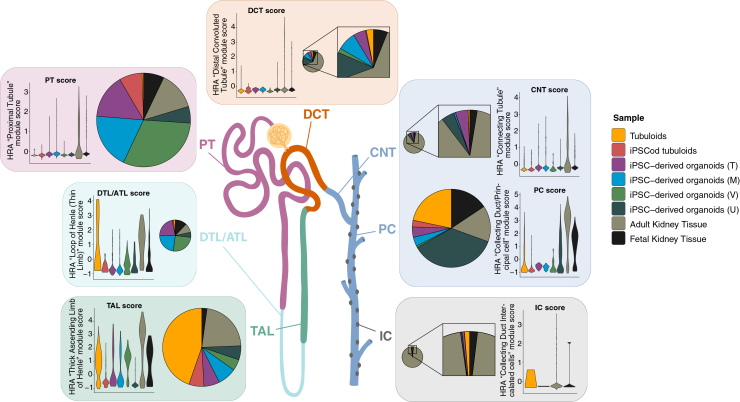
Visual representation of nephron epithelial cell type abundance and maturation in advanced *in vitro* kidney models The size of the pie charts relates to the total number of cells contributing to each population, and the violin plots show a “maturation score” per segment based on the expression of validated segment-specific markers.

To give a comprehensive overview of the main findings of this study, Figure 6 illustrates the approximate proportions and transcriptomic similarity between samples in each nephron epithelial segment. Despite the lack of complete maturation, iPSC-derived organoids derived using Takasato, Morizane, and Vanslambrouck’s protocols may be the most suitable for studies involving PT functionality. DTL/ATL cells are both more predominant and have a higher identity score in the original organoid protocols. For TAL-related investigations, tubuloids and Vanslambrouck organoids might be preferred. DCT and CNT cells are scarce in all models, but PCs are highly found in both tubuloids and Uchimura’s organoids, with a similar ability to replicate physiological markers. Lastly, albeit at very low numbers, tubuloids are the only model able to retain mature ICs. Future studies should aim for the enrichment of this cell population in the cultures.

This discussion cannot encompass all differentiating factors of the models examined in this study, but the data presented offer a detailed transcriptomic comparison, providing researchers with a resource to explore specific areas of interest. In general, each culture model has unique advantages, but our findings also indicate room for further refinement of the *in vivo*-like (re-)differentiation of various cell types within these advanced *in vitro* models. Some models are able to acquire expression of important functional markers, but there are still numerous genes relevant for kidney physiology that are virtually absent in the *in vitro* models selected in this study, including the PT sodium/glucose co-transporter encoded by *SLC5A2* (SGLT2), the TAL marker *UMOD*, the DCT transporter *SLC12A3* (NCC), and the CNT transporter *SLC8A1* (NCX1).

Lastly, it should be noted that we investigated the current state-of-the art kidney *in vitro* models for gene expression-based applications only. When selecting a culture model, other factors such as model morphology, interaction with other cell types (e.g., endothelium and stroma), cell polarity, and apical-basolateral access should also be considered, depending on the research objectives. It is also essential to recognize that mRNA expression may not always correlate with protein expression or functionality, warranting careful interpretation of the data.

In conclusion, through the parallel transcriptomic characterization of state-of-the-art kidney models, this study provides foundational understanding and a basis for choosing the most suitable model for particular applications. In this choice, not only a rudimentary differentiation direction at the population level is important but also the effective number of cells with a particular phenotype and the degree of differentiation or even the degree of expression of a specific gene. Examples include research on a specific genetic kidney disease or application of tubuloid/organoid cells for a specific transport function in a bioartificial organ. For research on development or, on the contrary, regeneration, it is important to be informed about the maturity and adaptive or non-adaptive phenotype of the cells in addition to the direction of differentiation.

### Limitations of the study

This study has limitations. First, the fact that low cell numbers had to be extracted from all samples to accommodate for the number of sequenced tubuloid cells. The small starting sample size and the subsequent subclustering rounds might have affected cell diversity and power in our analyses, making our assumptions on cellular proportions tentative. For example, we could not capture ICs from Uchimura’s organoids, while these have been previously shown to contain cells with apical ATP6V1B1 protein staining.^7^ Second, despite trying to capture as much epithelium as possible, the subsetting of cells by *EPCAM* positivity might have caused a biased cell type selection. EPCAM is considered a broad epithelial marker; however, it is not equally expressed by all kidney tubular epithelial cells.^73^ When comparing *EPCAM* expression across cell types in the adult kidney tissue dataset, we could confirm that PT cells had lower abundance compared to other tubular cell types (Figure S7A). Low percentages of cells expressing *EPCAM* were especially observed for damaged PT (dPT) (17.53%) and healthy PT-S1/2 (30.64%) clusters (Figure S7B), which might have affected the abundance of these cell types in our study. Third, our analyses lack validation at the protein/functional level. Although functional characterization of some genes presented here has been reported by others in kidney organoids obtained using the protocols included,^67^^,^^74^^,^^75^ future studies should further validate mature/identity marker expression in all models to confirm their maturity and modeling potential. Moreover, the pathways found enriched in each subpopulation are meant to serve as guidance but will require future experimental validation. Finally, it should be explored whether different iPSC lines have different maturation potential as that would influence our comparison of the protocols.

## Resource availability

### Lead contact

Requests for further information and resources should be directed to the lead contact, Hans Clevers (h.clevers@hubrecht.eu).

### Materials availability

This study did not generate new unique reagents.

### Data and code availability

• Single-cell RNA sequencing data have been deposited to the Gene Expression Omnibus (GEO) repository (GEO: GSE303210) and is publicly available.
• This paper also analyzes existing, publicly available data. All accession numbers can be found in Table 1.
• A web-based shinyapp has been generated for the free exploration of the integrated data: https://kidneymodelcomparison.shinyapps.io/shinyapp/.
• This paper does not report original code.
• Any additional information required to reanalyze the data reported in this paper is available from the lead contact upon request.

## Acknowledgments

We acknowledge the support of the partners of “Regenerative Medicine Crossing Borders” (RegMed XB), powered by 10.13039/100016036Health Holland, and the Gravitation Program “Materials Driven Regeneration,” funded by the 10.13039/501100003246Netherlands Organisation for Scientific Research (024.003.013). We also thank Katja Jansen for the scientific discussions.

## Author contributions

C.P.C. gathered the data, performed the analyses, and wrote the manuscript; C.P.C. and F.A.Y.Y. conceptualized the manuscript; F.A.Y.Y. and C.M.E.A. cultured and processed the iPSCod tubuloids to be sequenced; C.M.E.A., F.A.Y.Y., G.G.G.S., M.B.R., M.C.V., and H.C. reviewed and edited the manuscript.

## Declaration of interests

H.C. is the inventor of several patents related to organoid technology. H.C.’s full disclosure is given at https://www.uu.nl/staff/JCClevers/.

## STAR★Methods

### Key resources table

**Table:** 

REAGENT or RESOURCE	SOURCE	IDENTIFIER
**Biological samples**		

iPSC-15 for the generation of iPSCod tubuloids	Radboud University Medical Center (Nijmegen)	N/A

**Chemicals, peptides, and recombinant proteins**		

Cultrex Basement Membrane Extract (BME), Growth Factor Reduced, Type 2	R&D Systems, Bio-Techne	3533-001-02
Advanced DMEM-F12	Thermo Fisher Scientific	12634-010
Penicillin/Streptomycin	Thermo Fisher Scientific	15140122
HEPES	Thermo Fisher Scientific	15630056
GlutaMax	Thermo Fisher Scientific	35050038
R-spondin 3-conditioned medium	U-Protein Express	Custom order
B-27 supplement	Thermo Fisher Scientific	17504044
Human EGF	Peprotech	AF-100-15
FGF10	Peprotech	100-26
A83-01	Tocris	2939
N-acetyl-L-cysteine	Sigma-Aldrich	A9165
Y-27632 dihydrochloride	AbMole	M1817
Forskolin	Tocris	1099
Fludrocortisone acetate	Sigma-Aldrich	F0180000
Dispase II	Thermo Fisher Scientific	17105041
Accutase	Thermo Fisher Scientific	00-4555-56

**Deposited data**		

Raw and processed single-cell RNA sequencing data of differentiated iPSCod tubuloids	Gene expression omnibus (GEO) https://www.ncbi.nlm.nih.gov/geo/	GEO: GSE303210

**Software and algorithms**		

R (v.4.4.1)	https://www.r-project.org	N/A
RStudio	https://rstudio.com/	N/A
Seurat (v.5.2.1)	Hao et al.^76^	https://github.com/satijalab/seurat
NESTscore (v.1.0.0)	Naas et al.^78^	https://github.com/jn-goe/NESTScore
ComplexHeatmap (v.2.20.0)	Gu et al.^80^	https://github.com/jokergoo/ComplexHeatmap
clusterProfiler (v.4.12.6)	Wu et al.^82^	https://github.com/YuLab-SMU/clusterProfiler
ShinyCell2 (v.1.0.0)	Chen et al.^85^	https://github.com/the-ouyang-lab/ShinyCell2
Adobe Illustrator	Adobe Inc.	N/A
Adobe InDesign	Adobe Inc.	N/A

### Experimental model and study participant details

#### iPSCod tubuloid generation and culture

iPSCod tubuloids were generated as described by Yousef Yengej et al. (2023) using iPSC- 15-0001. Permission for the creation and use of iPSCs was obtained from the local ethics commission for human-related research of the Radboud University Medical Center (the Netherlands) (approval n°: 2015-1543 and 2006-048).^16^ For their expansion, iPSCod tubuloids were cultured in basement membrane extract (BME, R&D Systems) domes in expansion medium (EM), which consisted of ADF+++ (Advanced DMEM-F12 with 1% penicillin/streptomycin, 1% HEPES, 1% GlutaMAX (all from Thermofisher Scientific)) supplemented with 1% Rspondin 3-conditioned medium (U-Protein Express), 1.6% B27-supplement (Thermofisher Scientific), 50 ng/mL EGF (Peprotech), 100 ng/mL FGF-10 (Peprotech), 1 mM N-acetyl-L-cysteine (Sigma), 5 μM A83-01 (Tocris), and 10 μM Y-27632 (Abmole). Differentiation was achieved by treatment with FAF medium (10 μM forskolin (Tocris), 5 μM A83-01 (Tocris), and 10 μM fludrocortisone acetate (Sigma) in ADF+++) for 7 days.^15^

### Method details

#### iPSCod tubuloid 10× genomics sequencing

Upon completion of the differentiation treatment, iPSCod tubuloids were recovered by addition of 1 mg/mL dispase II (Thermofisher Scientific) to the medium and incubation for 30 min at 37°C. Tubuloids were subsequently dissociated by treatment with Accutase (Thermofisher Scientific) and mechanical shearing using a flame-polished Pasteur pipette. After washing with PBS, the cell suspension was fixed by adding frozen methanol dropwise. The sample was thereafter stored at −80°C until further processing. Library preparation and 10× genomics sequencing were carried out by Single Cell Discoveries (Utrecht, The Netherlands).^15^

### Quantification and statistical analysis

#### Processing of iPSCod tubuloid scRNAseq data

Mapping of sample reads to the human genome GRCh38 was performed using Cell Ranger (10× Genomics). The subsequent data processing was carried out using the Seurat package (v.5.2.1)^76^ in R (v.4.4.1).

#### Public dataset collection and integration

The datasets compared in this study can be found in Table 1. From the tubuloid scRNAseq dataset we exclusively selected FAF-differentiated cells. Similarly, from both iPSC-derived organoid datasets we only included differentiated samples (final time point). From the fetal kidney tissue dataset only the week 16 time point was used. Seurat objects for each dataset were created using features expressed in at least 5 cells.

To improve comparability and reduce batch effects all datasets were all downsampled to include the same number of cells as the dataset with the lowest cell count (tubuloids, with 2,528 cells). Moreover, since tubuloids and iPSCod tubuloids mostly contain epithelial cells, we subsetted epithelial cells from all other datasets based on positive (>0) *EPCAM* expression prior to downsampling. As quality control, cells containing less than 300 transcripts were filtered out from all datasets. Upper cuts were adapted to each sample based on feature count distribution: 7,500 features for the tubuloid dataset; 8,000 for the iPSCod tubuloid dataset; 9,000 for the Vanslambrouck dataset; and 6,000 for the fetal kidney tissue dataset. The human kidney tissue, Takasato and Morizane, and Uchimura datasets had already been processed^7^^,^^17^^,^^19^ and thus, quality control measures were not re-applied. The tubuloid and Takasato and Morizane organoid datasets were further filtered based on percent.mito<15. Median percentage of mitochondrial gene expression per cell across samples was below 10%, except for adult kidney tissue (<25%). Extensive QC metrics can be found in Table S1.

Integration of all datasets, including iPSCod tubuloids, was performed using Seurat’s reciprocal principal component analysis (RPCA) with default parameters.^77^ Clusters were determined using 1:40 dimensions and a resolution of 2.2, and DEGs per cluster were found using the *FindAllMarkers* function (logfc.threshold = 0.25). At this high resolution we retrieved 33 clusters, however 2 clusters were merged to compose LOH/CD; 5 for LOH/DCT; 5 for CNT/PC; 4 for cycling; 5 for PT; and 3 for stromal-like cells. Ultimately, we identified 15 major clusters.

#### Subclustering analyses

Using the adult kidney tissue reference metadata and by computing DEGs per cluster, we were able to assign broad identities to each cell population. Despite prior selection of epithelial cells, some non-epithelial cell types (e.g., endothelium and stromal-like) and non-kidney cell types (labeled as off-target) were observed. Consequently, subclustering was performed to exclusively look at the main kidney tubular epithelium populations (i.e., clusters PT, LOH/DCT, LOH/CD, CNT/PC, and IC). Upon subsetting, the data was rescaled, and new clusters were obtained using 1:35 dims and res = 2. Since some populations could still not be separately clustered (e.g., TAL/DCT), an additional round of subsetting was performed on the major tubular segment clusters (i.e., PT, TAL/DCT, and CNT/PC) for an in-depth exploration of cell types. After subsetting each population, the data was rescaled and the top 500 variable features were used for PCA. Clustering was achieved using the Leiden algorithm and different settings per subset (PT: dims = 1:25, res = 1.2; TAL/DCT: dims = 1:20, res = 1.3; CNT/PC: dims = 1:25, res = 0.8), and markers per cluster were identified using *FindAllMarkers* with logfc.threshold = 0.25 and min.pct = 0.2.

#### Batch effects assessment

To explore batch effects or the unequal distribution of samples upon integration we used the recently developed tool NEighborhood Sample homogeneiTy (NEST)-Score^78^ using k_nn = 30 and ndims = 40. Additionally, we computed the k-nearest neighbor Batch Effect Test (kBET) acceptance rate in the tubular epithelium subset using the first 20 dimensions, k0 = 10, and testSize = 0.1.^79^

#### Differential expression and pathway enrichment analyses

In order to compute the top 25 DEGs specific for each major tubular cell type, we first subsetted adult kidney-derived cells from the tubular epithelium atlas (Figure 1D). Top 25 PT/aPT markers (pct.1 > 0.3, avg_log2FC > 2, p_val_adj<1E−10) could be directly acquired using the clusters.l2 annotations. However, for the other four major cell types (TAL, DCT, CNT, and PC), we first had to transfer clusters.l3 annotations from each segment, which better separated all cell types, to the tubular epithelium object. *FindMarkers* (using the default Wilcoxon Rank-Sum test) was thereafter used to identify top DEGs in each specific nephron cell type exclusively based on the adult kidney tissue dataset. The top 25 TAL-specific DEGs were based on TAL, TAL2, and aTAL cells (pct.1 > 0.4, avg_log2FC > 1.5, p_val_adj<1E−10) and plotted across all (a)TAL populations. For DCT, top 25 specific genes were computed from both DCT and dDCT (pct.1 > 0.3, avg_log2FC > 1.5, p_val_adj<1E−10). Lastly, PC and CNT top 25 DEGs were taken from cells in the PC (pct.1 > 0.5, avg_log2FC > 2, p_val_adj<1E−10) and CNT (pct.1 > 0.35, avg_log2FC > 1.5, p_val_adj<0.01) clusters, respectively. However, given that a cluster contained a mix of both (CNT/PC), we plotted both lists across all PC clusters, CNT/PC and CNT cells. ComplexHeatmap (v.2.20.0)^80^ was used to plot these top 25 gene lists.

To make lists of genes enriched only in mature cells for each cell type, we again transferred all clusters.l3 annotations to the tubular epithelium atlas, subsetted the adult kidney tissue dataset, and used *FindMarkers* for either PT-S1/2, TAL and TAL2, DCT, or PC (avg_log2FC > 1, p_val_adj<0.05). Genes in each list were subsequently manually distributed into categories (transporters, enzymes, others, or transcription factors) (up to 20 genes per category). PT-S1/2 DEGs were plotted across all (a)PT populations; TAL(2) DEGs across (a)TAL clusters only; DCT DEGs across DCT and dDCT clusters; and PC DEGs across PC populations only (no CNT/PC, CNT, or tPC/IC-IC). Specific lists for mature CNT cells are not shown because the vast majority of genes were already present in the top 25 list.

To explore pathways enriched in each epithelial cell cluster, we used the lists of positive DEGs per population in the integrated dataset (avg_log2FC > 1 and p_val_adj<0.05). Computation and rendering of enriched pathways based on MSigDb hallmark terms was performed using msigdbr (v.10.0.1),^81^ clusterProfiler (v.4.12.6),^82^ DOSE (v.3.30.5),^83^ and enrichplot (v.1.24.4).^84^ For multiciliated and hypoxic PT populations, gene set enrichment analysis was based on gene ontology (GO) terms.

#### Correlation analyses

Correlation in average expression between the models in each cell population was computed using the top 1000 variable genes to prevent variability driven by differences in sequencing depth. Correlation among TAL cells was explored taking into account all (a)TAL clusters, while for the correlation in DCT cells we exclusively subsetted DCT and dDCT. Correlations in expression between CNT and PC cells were explored using all PC and CNT clusters together, given that the mixed CNT/PC cluster could not be separated.

#### Module scores computation

Segment-specific module scores per sample were calculated using Seurat’s *AddModuleScore* function using validated markers available in the Human Reference Atlas’ Anatomical Structures, Cell Types, and Biomarkers (ASCT+B) tables.^45^

In the sensitivity analysis, we computed module scores using the top 25 DEGs lists per generated in the original analysis (see above). Additionally, top 25 DTL/ATL and IC DEGs were acquired using clusters.l2 annotations (DTL/ATL: pct.1 > 0.25, avg_log2FC > 1.5, p_val_adj<1E−10; IC: pct.1 > 0.3, avg_log2FC > 2, p_val_adj<1E−10).

#### ShinyApp generation

The web-based data explorer for the subsetted tubular epithelium data was generated using ShinyCell2.^85^

## Supplemental information

Document S1. Figures S1–S7, Tables S1 and S2, and supplemental references

Data S1. Cell numbers and DEGs per cluster in the initial atlas (clusters.l1), related to Figure 1

Data S2. Cell numbers and DEGs per cluster in the subsetted tubular epithelium atlas (clusters.l2), related to Figure 1

Data S3. Cell numbers and DEGs per cluster in the subsetted PT population (PT clusters.l3), related to Figure 2

Data S4. Cell numbers and DEGs per cluster in the subsetted TAL/DCT population (TAL/DCT clusters.l3), related to Figure 3

Data S5. Cell numbers and DEGs per cluster in the subsetted CNT/PC population (CNT/PC clusters.l3), related to Figure 4
