# Supplementary material for: Single-cell transcriptomic comparison of tubular segment maturation in advanced human in vitro kidney models
Source: iScience. 2026 May 22;29(6):116053. doi: 10.1016/j.isci.2026.116053 (PMC13223991; doi:10.1016/j.isci.2026.116053)
Supplement: Document S1. Figures S1–S7, Tables S1 and S2, and supplemental references [file mmc1.pdf]

iScience, Volume *29*

Supplemental information

Single-cell transcriptomic comparison of tubular segment maturation in advanced human *in vitro* kidney models

Carla Pou Casellas, Fjodor A. Yousef Yengej, Carola M.E. Ammerlaan, Gisela G. Slaats, Maarten B. Rookmaaker, Marianne C. Verhaar, and Hans Clevers

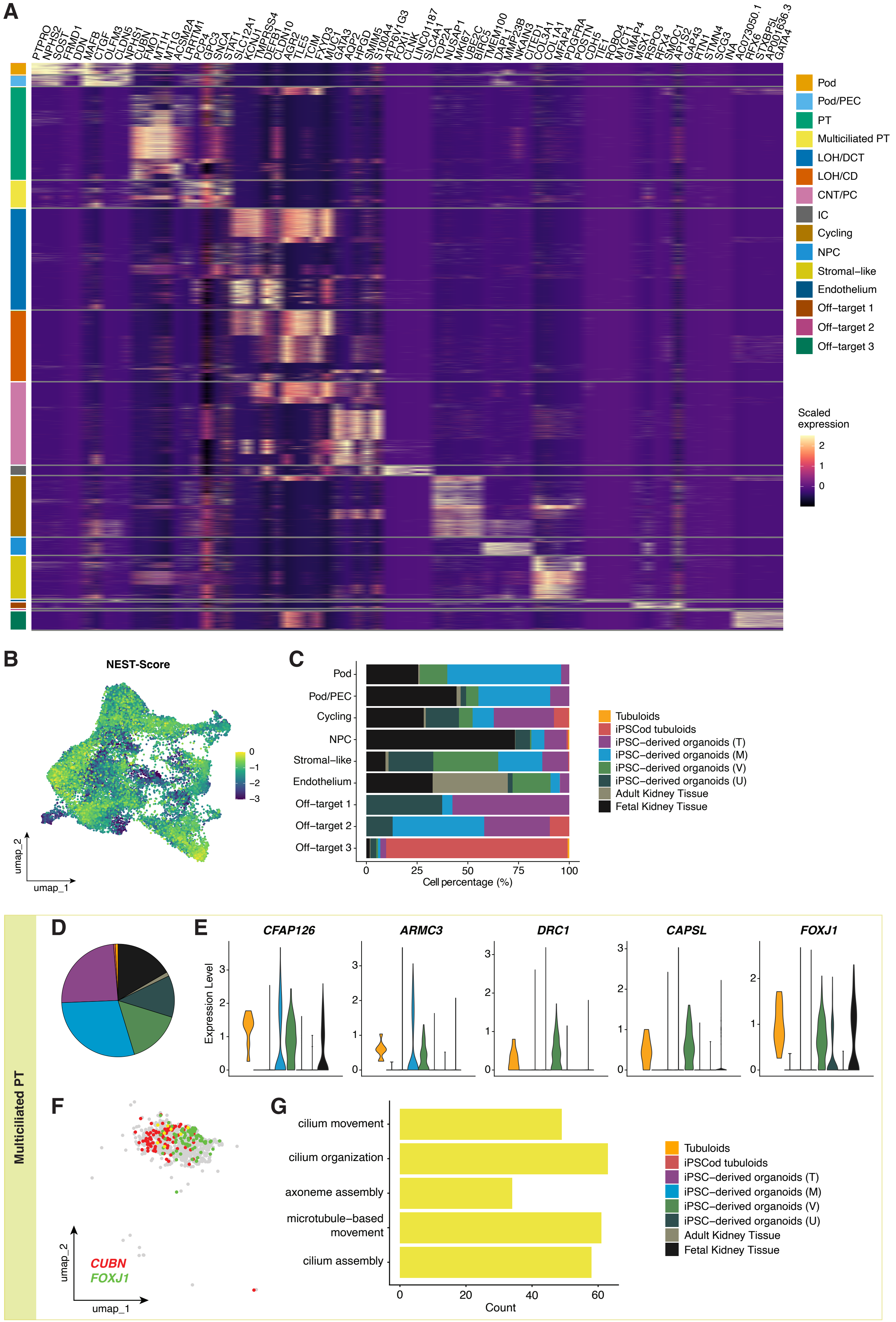

Figure S1. Cell populations found in the advanced *in vitro* model cell atlas, Related to Figure 1. (A) Heatmap depicting top 5 differentially expressed genes in each cluster. (B) NEST-Scores indicating good (~0) sample mixing or a sam-

ple-biased distribution (<0). (C) Cell percentage contribution from each sample to non-tubular epithelium populations. (D) Pie chart showing sample proportions in the multiciliated PT cluster. (E) Violin plots of top multiciliated PT markers across samples. (F) Feature plot showing co-expression of *CUBN* and *FOXJ1* in multiciliated PT cells. (G) GO term enrichment analysis in the multiciliated PT cluster.

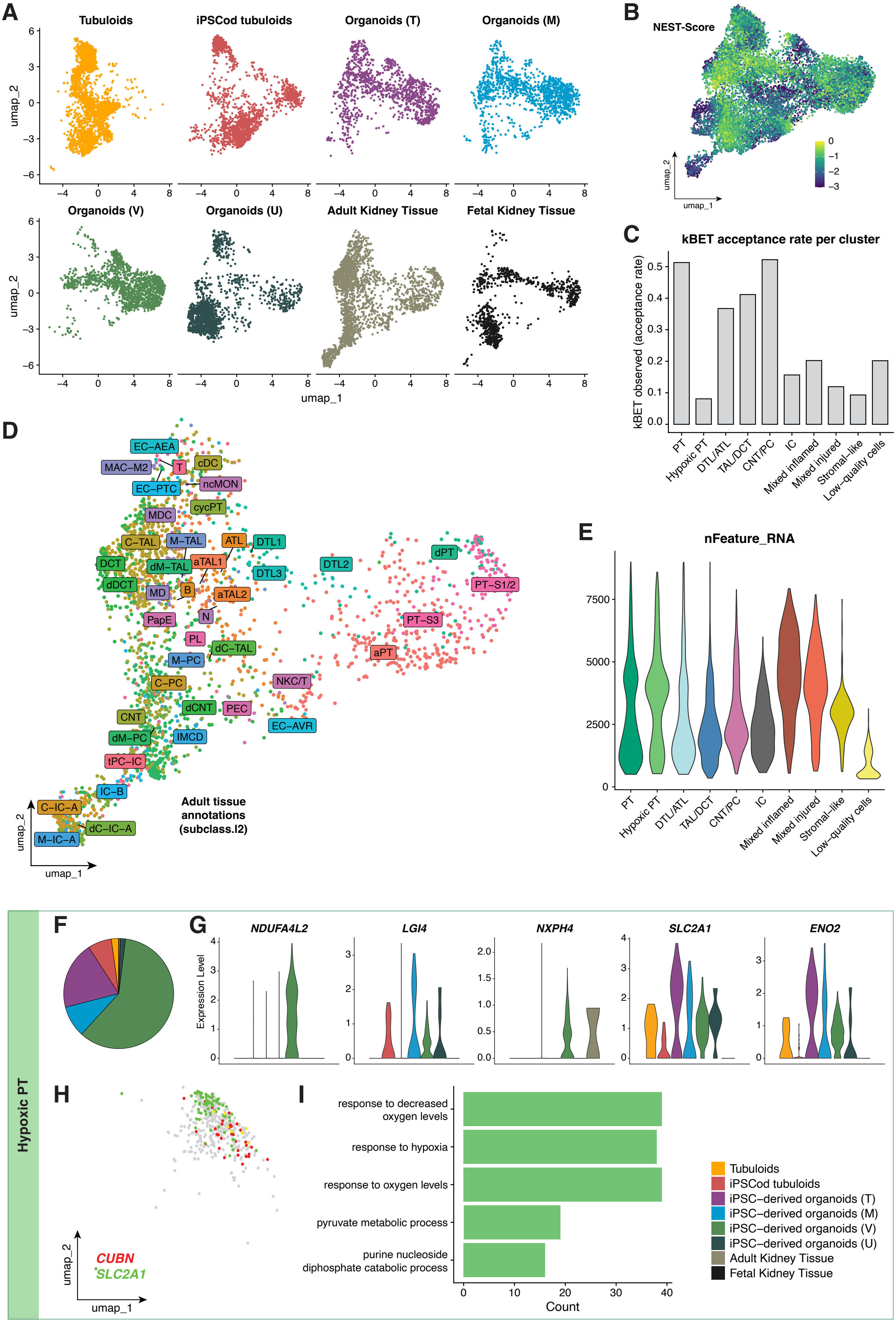

Figure S2. Cell populations found in the sub-clustered tubular epithelium atlas, Related to Figure 1. (A) UMAP plot of the sub-clustered tubular epithelium split by sample. (B) NEST-Scores indicating good (~0) sample mixing or a sample-biased distribution (<0). (C) kBET acceptance rate per cluster, where higher values indicate higher sample mixing. (D) UMAP plot of the sub-clustered atlas depicting the original annotations of adult kidney tissue-derived cells (subclass.l2 from Lake et al. (2023) [1]). (E) Violin plot with feature counts per cluster. (F) Pie chart showing sample proportions in the hypoxic PT cluster. (G) Violin plots of top hypoxic PT markers across samples. (H) Feature plot showing co-expression of *CUBN* and *SLC2A1* in hypoxic PT cells. (I) GO term enrichment analysis in the hypoxic PT cluster.

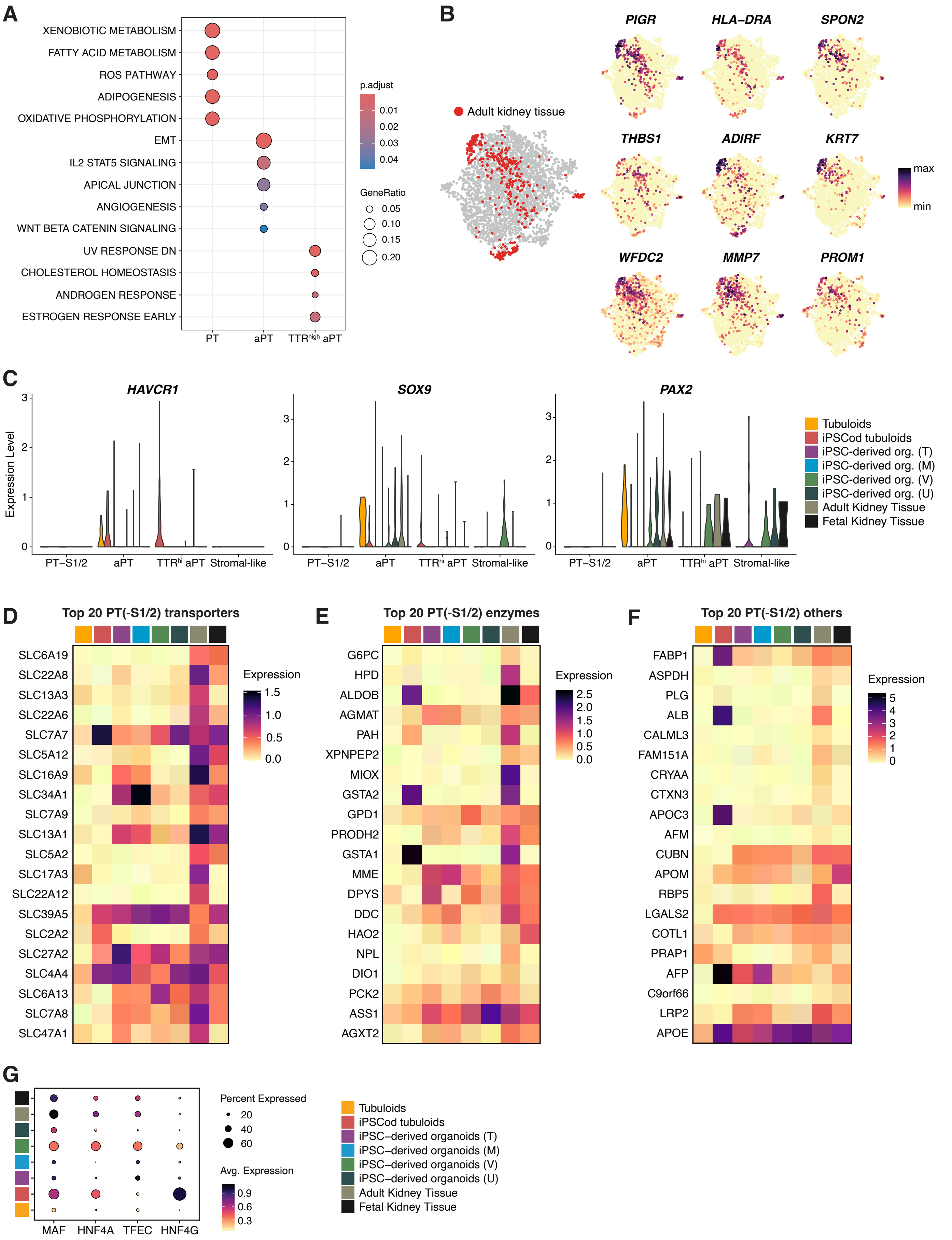

Figure S3. PT cell markers across advanced *in vitro* models and primary kidney tissue, Related to Figure 2. (A) Gene set enrichment analysis (MSigDb) in (a)PT populations. (B) Feature plots of markers expressed in adult kidney tissue-derived aPT cells. (C) Violin plots showing expression of common PT injury markers across samples in each (a)PT cluster. (D-F) Heatmaps of the top transporters, enzymes, and other markers (up to 20) differentially expressed in mature PT(-S1/2) plotted across samples. (G) Dot plot showing expression of top PT TFs across samples.

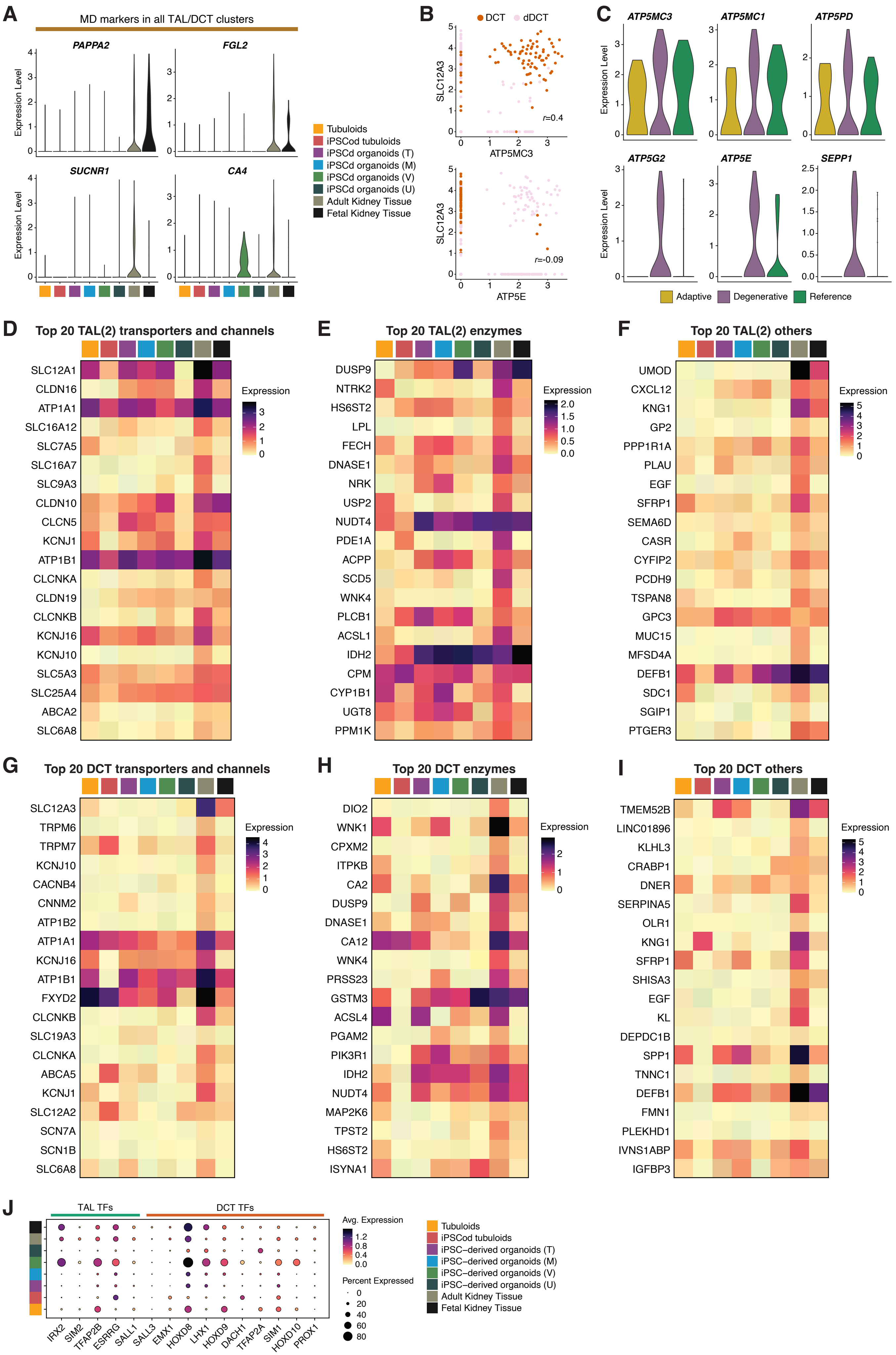

A
MD markers in all TAL/DCT clusters
PAPPA2
FGL2
SUCNR1
CA4
Expression Level
Tubuloids
iPSCod tubuloids
iPSCd organoids (T)
iPSCd organoids (M)
iPSCd organoids (V)
iPSCd organoids (U)
Adult Kidney Tissue
Fetal Kidney Tissue
B
DCT
dDCT
SLC12A3
ATP5MC3
r=0.4
ATP5E
r=-0.09
C
ATP5MC3
ATP5MC1
ATP5PD
ATP5G2
ATP5E
SEPP1
Adaptive
Degenerative
Reference
D
Top 20 TAL(2) transporters and channels
SLC12A1
CLDN16
ATP1A1
SLC16A12
SLC7A5
SLC16A7
SLC9A3
CLDN10
CLCN5
KCNJ1
ATP1B1
CLCNKA
CLDN19
CLCNKB
KCNJ16
KCNJ10
SLC5A3
SLC25A4
ABCA2
SLC6A8
Expression
E
Top 20 TAL(2) enzymes
DUSP9
NTRK2
HS6ST2
LPL
FECH
DNASE1
NRK
USP2
NUDT4
PDE1A
ACPP
SCD5
WNK4
PLCB1
ACSL1
IDH2
CPM
CYP1B1
UGT8
PPM1K
F
Top 20 TAL(2) others
UMOD
CXCL12
KNG1
GP2
PPP1R1A
PLAU
EGF
SFRP1
SEMA6D
CASR
CYFIP2
PCDH9
TSPAN8
GPC3
MUC15
MFSD4A
DEFB1
SDC1
SGIP1
PTGER3
G
Top 20 DCT transporters and channels
SLC12A3
TRPM6
TRPM7
KCNJ10
CACNB4
CNNM2
ATP1B2
ATP1A1
KCNJ16
ATP1B1
FXYD2
CLCNKB
SLC19A3
CLCNKA
ABCA5
KCNJ1
SLC12A2
SCN7A
SCN1B
SLC6A8
H
Top 20 DCT enzymes
DIO2
WNK1
CPXM2
ITPKB
CA2
DUSP9
DNASE1
CA12
WNK4
PRSS23
GSTM3
ACSL4
PGAM2
PIK3R1
IDH2
NUDT4
MAP2K6
TPST2
HS6ST2
ISYNA1
I
Top 20 DCT others
TMEM52B
LINC01896
KLHL3
CRABP1
DNER
SERPINA5
OLR1
KNG1
SFRP1
SHISA3
EGF
KL
DEPDC1B
SPP1
TNNC1
DEFB1
FMN1
PLEKHD1
IVNS1ABP
IGFBP3
J
TAL TFs
DCT TFs
IRX2
SIM2
TFAP2B
ESRRG
SALL1
SALL3
EMX1
HOXD8
LHX1
HOXD9
DACH1
TFAP2A
SIM1
HOXD10
PROX1
Avg. Expression
Percent Expressed
Tubuloids
iPSCod tubuloids
iPSC−derived organoids (T)
iPSC−derived organoids (M)
iPSC−derived organoids (V)
iPSC−derived organoids (U)
Adult Kidney Tissue
Fetal Kidney Tissue

Figure S4. TAL/DCT cell markers across advanced *in vitro* models and primary kidney tissue, Related to Figure 3. (A) Violin plots of top MD markers in all TAL/DCT clusters. (B) Scatter plot of expression levels of *SLC12A3* and either *ATP-5MC3* or *ATP5E* in DCT and dDCT cells. r indicates the Pearson correlation coefficient. (C) Violin plots of the expression of DCT and dDCT markers in each adult kidney cell state (adaptive, degenerative, or reference cells). (D-F) Heatmaps of the top transporters, enzymes, and other markers (up to 20) differentially expressed in mature TAL and TAL2 cells plotted across samples. (G-I) Heatmaps of the top transporters, enzymes, and other markers (up to 20) differentially expressed in mature DCT cells plotted across samples. (J) Dot plot showing expression of top TAL and DCT TFs across samples.

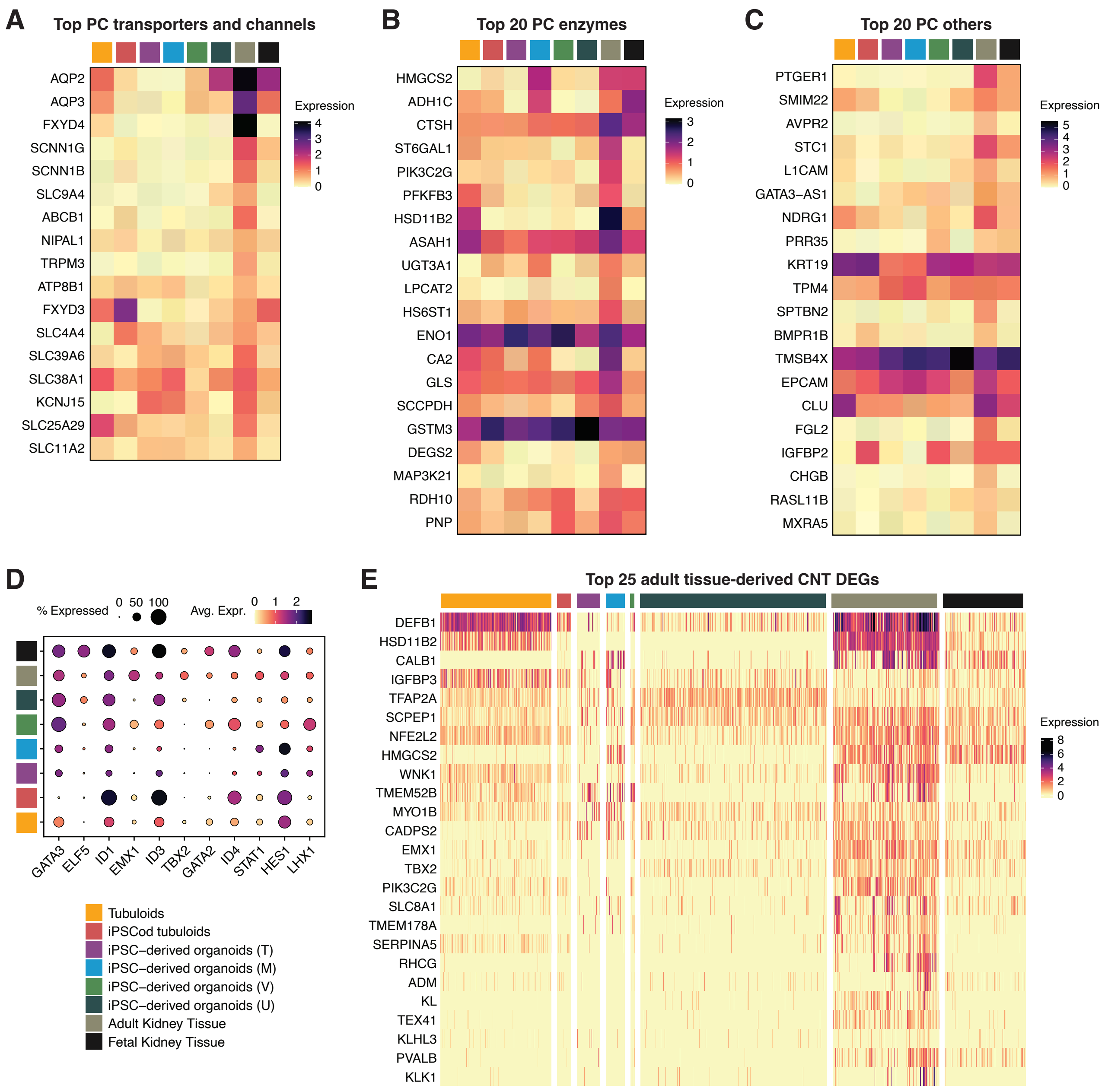

Figure S5. CNT/PC markers across advanced *in vitro* models and primary kidney tissue, Related to Figure 4. (A-C) Heatmaps of the top transporters/channels, enzymes, and other markers (up to 20) differentially expressed in mature PC plotted across samples. (D) Dot plot showing expression of top PC TFs across samples. (E) Heatmap of the top 25 DEGs in adult tissue-derived CNT plotted across samples.

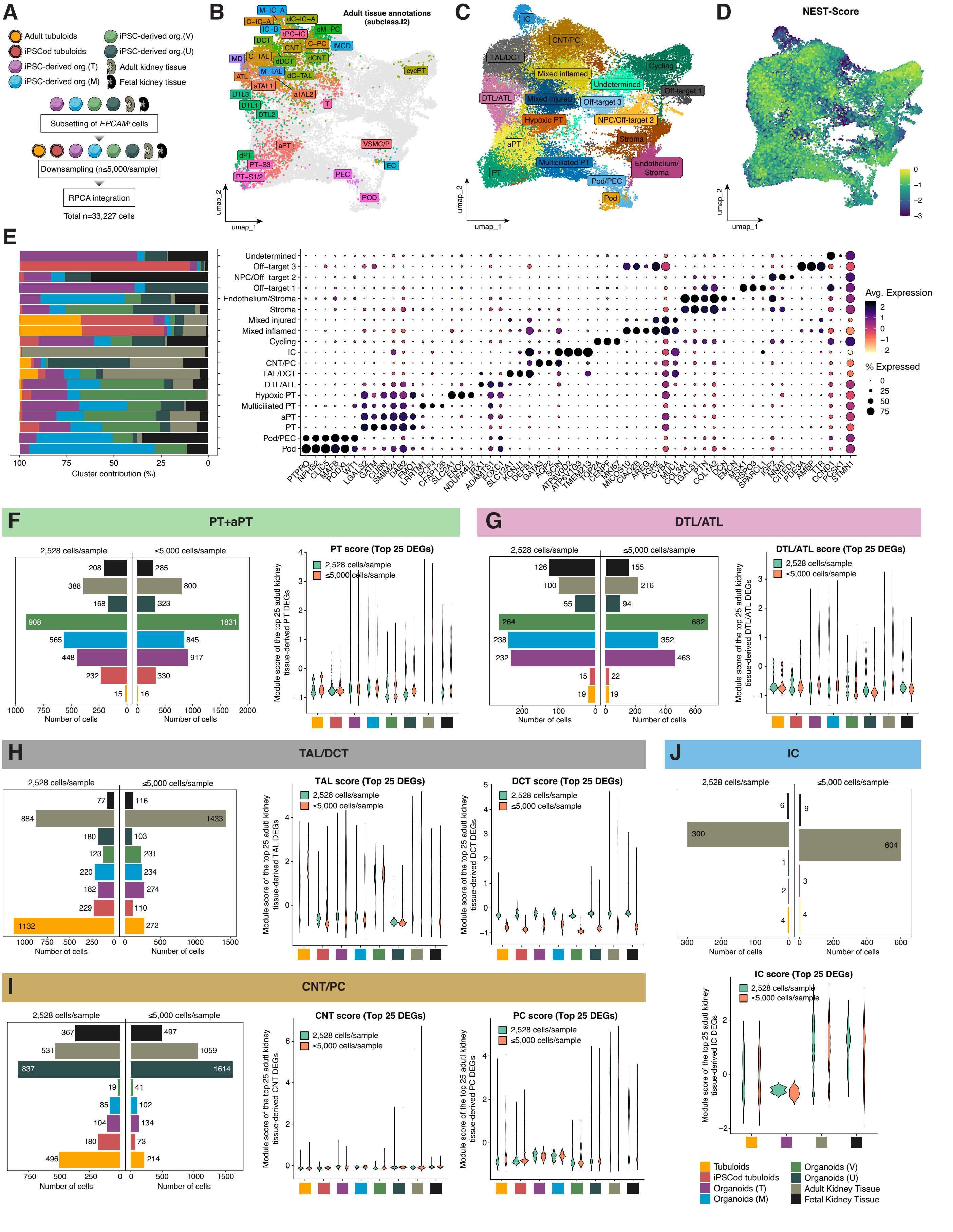

Figure S6. A sensitivity analysis with capped downsampling confirms the observations of the original analysis. (A) Diagram illustrating the processing strategy for integration of the datasets. (B) UMAP plot of the integrated atlas depicting the original annotations of adult kidney tissue-derived cells (subclass.l2 from Lake et al. (2023) [1]). (C) UMAP plot of the integrated atlas showing identified clusters. (D) NEST-Scores indicating good (~0) sample mixing or a sample-biased distribution (<0). (E) Dot plot depicting top 3 DEGs per cluster, including the cell contribution of each sample to the individual clusters. (F-J) Comparison of cell counts and "maturity" score per sample between the original and the capped downsampling strategy in PT+aPT (F), DTL/ATL (G), TAL/DCT (H), CNT/PC (I), and ICs (J). Cell type-specific scores were based on the expression of the top 25 DEGs per cell type in adult kidney tissue.

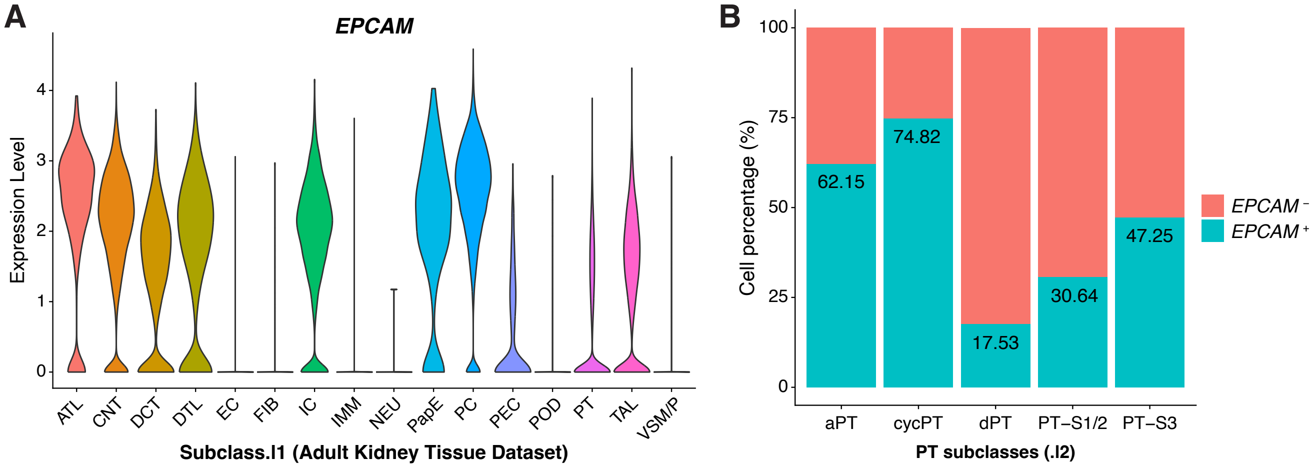

Figure S7. ***EPCAM* expression across kidney cell types.** (A) Violin plot depicting *EPCAM* expression in each cell cluster (adult kidney tissue dataset only). (B) Percentages of *EPCAM*+ cells in each PT subclass.

**Table S1. Quality control (QC) and cell number metrics from each dataset.** Related to Figure 1.								
	**Adult Tubuloids**	**iPSCod tubuloids**	**iPSC-derived org. (T)**	**iPSC-derived org. (M)**	**iPSC-derived org. (V)**	**iPSC-derived org. (U)**	**Adult Kidney Tissue**	**Fetal Kidney Tissue**
**Sequencing depth (features)**	36,601	24,059	24,572	24,572	23,287	19,880	28,136	19,707
**Initial range of genes/cell (median)**	[399-8,844] (3,000)	[289-10,576] (3,921)	[493-3,496] (984)	[493-3,499] (919)	[71-11,732] (3,772.5)	[1,001-3,999] (1,928)	[501-4,999] (1,276)	[42-8,094] (1,603)
**Range of genes/cell after QC + down-sampling (median)**	[551-7,447] (3,451)	[319-7,985] (3,922)	[501-3,492] (1,343.5)	[498-3,499] (1,241)	[484-8,992] (4,671)	[1,001-3,998] (2,395)	[501-4,996] (1,969)	[742-5,638] (1,944)
**Median UMI counts/cell after QC + down-sampling**	11,887	13,110	2,278	2,024	20,706	6,751.5	5,169.5	5,150
**Max. % mitochondrial genes after QC + down-sampling (median)**	15 (7.5)	84.1 (4.3)	15 (5.0)	15 (6.7)	50 (5.8)	15 (6.5)	50 (22.4)	43.6 (3.2)
**Cell numbers**								
**Initial n of cells**	3,339	4,248	46,270	25,120	16,412	16,938	109,741	8,503
**N of cells after QC (% of initial cells)**	2,528 (75.7%)	4,192 (98.7%)	45,433 (98.2%)	23,929 (95.2%)	10,907 (66.5%)	16,938 (100%)*	109,741 (100%)*	8,464 (99.5%)
**N of cells after *EPCAM+* subsetting (% of cells after QC)**	2,528 (100%)*	4,192 (100%)*	12,302 (27.1%)	3,606 (15.1%)	6,696 (42%)	4,443 (26.2%)	55,142 (50.2%)	3,458 (40.8%)
**N of cells after down-sampling (% of all *EPCAM+* cells)**	2,528 (100%)*	2,528 (60.3%)	2,528 (20.5%)	2,528 (70.1%)	2,528 (37.7%)	2,528 (56.9%)	2,528 (4.6%)	2,528 (73.1%)
**N of cells after tubular epithelium subsetting (% of all *EPCAM+* cells)**	2,507 (99.2%)	1,745 (41.6%)	1,157 (9.4%)	1,229 (34.1%)	1,628 (24.3%)	1,542 (34.7%)	2,441 (4.4%)	834 (24.1%)
*No subsetting performed								

Table S2. Cell number metrics from each dataset in the integrated atlas containing up to 5,000 cells/sample. Related to Figure S6.

	**Adult Tubuloids**	**iPSCod tubuloids**	**iPSC-derived org. (T)**	**iPSC-derived org. (M)**	**iPSC-derived org. (V)**	**iPSC-derived org. (U)**	**Adult Kidney Tissue**	**Fetal Kidney Tissue**
**N of cells after *EPCAM+* subsetting (% of cells after QC)**	2,528 (100%)	4,192 (100%)	12,302 (27.1%)	3,606 (15.1%)	6,696 (42%)	4,443 (26.2%)	55,142 (50.2%)	3,458 (40.8%)
**N of cells after down-sampling (% of all *EPCAM+* cells)**	2,528 (100%)	4,192 (100%)	5,000 (40.6%)	3,606 (100%)	5,000 (74.7%)	4,443 (100%)	5,000 (9.1%)	3,458 (100%)

Supplemental references

[1]. Lake, B.B., et al., An atlas of healthy and injured cell states and niches in the human kidney. Nature, 2023. 619(7970): p. 585-594.
